# Module evolution and substrate specificity of fungal nonribosomal peptide synthetases involved in siderophore biosynthesis

**DOI:** 10.1186/1471-2148-8-328

**Published:** 2008-12-03

**Authors:** Kathryn E Bushley, Daniel R Ripoll, B Gillian Turgeon

**Affiliations:** 1 Department of Plant Pathology & Plant-Microbe Biology, 334 Plant Science Building, Cornell University, Ithaca, NY, 14853, USA; 2 Computational Biology Service Unit, Life Sciences Core Laboratories Center, Cornell University, 621 Frank HT Rhodes Hall, Hoy Road, Ithaca, NY, 14853 3801, USA

## Abstract

**Background:**

Most filamentous ascomycete fungi produce high affinity iron chelators called siderophores, biosynthesized nonribosomally by multimodular adenylating enzymes called nonribosomal peptide synthetases (NRPSs). While genes encoding the majority of NRPSs are intermittently distributed across the fungal kingdom, those encoding ferrichrome synthetase NRPSs, responsible for biosynthesis of ferrichrome siderophores, are conserved, which offers an opportunity to trace their evolution and the genesis of their multimodular domain architecture. Furthermore, since the chemistry of many ferrichromes is known, the biochemical and structural 'rules' guiding NRPS substrate choice can be addressed using protein structural modeling and evolutionary approaches.

**Results:**

A search of forty-nine complete fungal genome sequences revealed that, with the exception of *Schizosaccharomyces pombe*, none of the yeast, chytrid, or zygomycete genomes contained a candidate ferrichrome synthetase. In contrast, all filamentous ascomycetes queried contained at least one, while presence and numbers in basidiomycetes varied. Genes encoding ferrichrome synthetases were monophyletic when analyzed with other NRPSs. Phylogenetic analyses provided support for an ancestral duplication event resulting in two main lineages. They also supported the proposed hypothesis that ferrichrome synthetases derive from an ancestral hexamodular gene, likely created by tandem duplication of complete NRPS modules. Recurrent losses of individual domains or complete modules from this ancestral gene best explain the diversity of extant domain architectures observed. Key residues and regions in the adenylation domain pocket involved in substrate choice and for binding the amino and carboxy termini of the substrate were identified.

**Conclusion:**

Iron-chelating ferrichrome synthetases appear restricted to fission yeast, filamentous ascomycetes, and basidiomycetes and fall into two main lineages. Phylogenetic analyses suggest that loss of domains or modules led to evolution of iterative biosynthetic mechanisms that allow flexibility in biosynthesis of the ferrichrome product. The 10 amino acid NRPS code, proposed earlier, failed when we tried to infer substrate preference. Instead, our analyses point to several regions of the binding pocket important in substrate choice and suggest that two positions of the code are involved in substrate anchoring, not substrate choice.

## Background

Most filamentous ascomycete fungi produce high affinity iron chelator siderophores for scavenging environmental iron and for cellular sequestration of reactive iron [1]. All known fungal siderophores are synthesized by nonribosomal peptide synthetases (NRPSs) [2], large, usually multimodular enzymes that catalyze peptide bond formation independent of ribosomes. NRPS modules consist of three core domains, ordered 5'A-T-C 3': 1) an adenylation (A) domain responsible for recognizing and activating a substrate molecule *via *adenylation with ATP, 2) a thiolation (T) domain which binds the substrate to the NRPS protein and 3) a condensation (C) domain which joins two substrates through a condensation reaction.

Although the number of NRPSs encoded by individual filamentous fungi varies from 0 to > 20, most of these and their corresponding metabolites are not conserved across the fungal kingdom, making it difficult to trace the evolutionary history of the corresponding genes. Various evolutionary processes may account for this. The observation that A-T-C modules from a single NRPS often group together as a monophyletic clade suggests tandem duplication of modules as a possible mechanism by which multimodular NRPSs arise [3]. It is clear, however, that other mechanisms such as recombination and gene conversion also operate [4]. Ferrichrome synthetases, which biosynthesize ferrichromes, fungal hydroxamate siderophores that function primarily in intracellular iron storage, are among the most conserved NRPSs, offering an opportunity to trace the evolutionary history of the corresponding genes across fungi.

**Figure 1:**
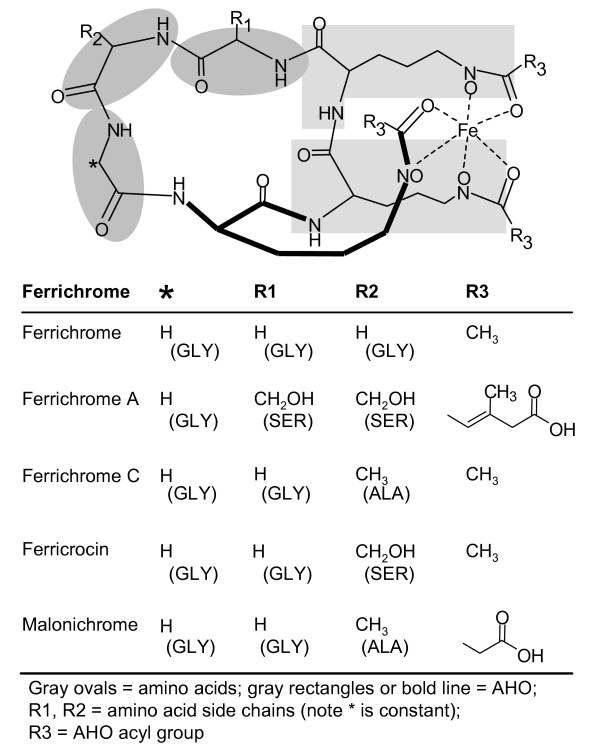
**Ferrichrome structure**. Chemical structure of five different ferrichromes and the corresponding amino acid and AHO acyl group constituents.

The chemical products of ferrichrome synthetases have been characterized for at least one member of the majority of Ascomycete and Basidiomycete orders [5,6]. This class of siderophore includes compounds such as ferricrocin, ferrichrome, ferrichrome A, ferrichrome C, and malonichrome. Most ferrichrome siderophores are cyclic hexapeptides [Fig. 1], with the exceptions of tetraglycylferrichrome, a cyclic heptapeptide, and desdiserylglycerylferrirhodin (DDF) a linear tripeptide of ornithine residues [7]. The chemical structure of ferrichromes is also conserved, consisting of six substrate molecules: a core heme-binding unit consisting of three *N*^5^-acyl-*N*^5^-hydroxy-L-ornithines (AHO) and a ring of three amino acids (Fig. 1). One amino acid is always a glycine, while the remaining two amino acids can be alanine, serine, or glycine [5,7]. Ferrichrome has three glycines, ferrichrome A has two serines and one glycine, ferrichrome C and malonichrome have two glycines and one alanine, and ferricrocin has two glycines and one serine [7]. Acyl groups attached to AHO substrates can also vary (Fig. 1).

Substrate specificity of NRPSs is believed to be mediated by the A domain [8-10] although some studies have suggested a role for the C domain in selective acceptance of substrates from the A domain [8,11]. A 10 amino acid (AA) NRPS substrate specificity "code" consisting of single, nonadjacent amino acid residues in the A domain has been proposed, based primarily on examination of bacterial NRPS A domains [9,12]. Few of these have been tested experimentally and the extent to which this code is applicable to fungal NRPS A domains remains unknown [13]. Since the chemical structure and composition of siderophores produced by fungal ferrichrome synthetases is largely conserved, phylogenetic and structural analyses of these proteins provide an opportunity to correlate protein structure and candidate specificity residues of the A domains with known chemical products.

Ferrichrome siderophores perform key functions in fungal cells. Early work on *Neurospora crassa *suggested that ferricrocin aids in asexual spore germination by storing iron reserves within spores [14,15]. This role in asexual development has been confirmed for ferrichrome-type siderophores of other fungal species such as *Penicillium chrysogenum *[16] and *Aspergillus nidulans *[17]. In contrast, *Cochliobolus heterostrophus *and *Fusarium graminearum *intracellular siderophores have a major role in sexual spore development, but no obvious role in asexual development [18]. A role in sexual development has also been described for intracellular siderophores of *A. nidulans *[19]. Intracellular siderophores are thought to buffer against reactive oxygen species (ROS) generated by the Haber-Weiss-Fenton reaction in the presence of unbound iron, by sequestering cellular free iron [16]. Indeed, *A. nidulans *mutants lacking ability to produce intracellular siderophores show increased levels of intracellular free iron [17] and a corresponding increase in sensitivity to ROS [19]. *C. heterostrophus *mutants lacking ability to make intracellular siderophores, however, are like wild-type (WT) strains in terms of sensitivity to ROS, although mutants lacking extracellular siderophores do show increased sensitivity to ROS [20].

These subtle functional differences observed between intracellular ferrichrome synthetase mutantsof *C. heterostrophus *and *A. nidulans*, as well as the presence of two or more copies of the genes encoding ferrichrome synthetases in some fungal species suggested the hypothesis that more than one lineage of *NPS *genes may be responsible for intracellular siderophore biosynthesis in fungi. In this study, we sought to: 1) identify homologs of *C. heterostrophus *and *A. nidulans *ferrichrome synthetases in a phylogenetically representative sample of fungal genomes, 2) address the hypothesis of two distinct lineages of ferrichrome synthetases 3) analyze the structural evolution of enzymatic domains encoded by these genes by phylogenetic analysis, and 4) investigate key positions in A domains that may be involved in substrate specificity.

## Methods

### Genomes surveyed for ferrichrome-associated nonribosomal peptide synthetases

Candidate homologs of *C. heterostrophus *NPS2 [3,18] and *A. nidulans *SidC [19] were identified through blastp and tblastn searches using individual A domains from both NPS2 and SidC proteins as a query set. Fungal genome datasets interrogated included those at the Broad Institute http://www.broad.mit.edu/ (*A. nidulans, Aspergillus terreus, Batrachochytrium dendrobatis, Botrytis cinerea, Candida albicans, Candida guilliermondii, Candida lusitaniae, Chaetomium globosum, Coccidioides immitis, Coprinus cinereus, Cryptococcus neoformans, F. graminearum, Histoplasma capsulatum, Magnaporthe grisea, N. crassa, Rhizopus oryzae, Sclerotinia sclerotiorum, Stagonospora nodorum, Uncinocarpus reesii, and Ustilago maydis*), the Sanger Institute (*Schizosaccharomyces pombe, Aspergillus fumigatus*), the Joint Genome Institute http://www.jgi.doe.gov/ (*Laccaria bicolor, Aspergillus niger, Trichoderma reesii*, *Phanerochaete chrysosporium*, and *Phycomyces blakesleeanus*), the DOGAN database http://www.bio.nite.go.jp/ngac/e/rib40-e.html (*Aspergillus oryzae*), and the raw genome sequence of *Alternaria brassicicola*, available at Washington University http://genome.wustl.edu. The all fungal blast portal at the Saccharomyces Genome Database http://seq.yeastgenome.org/cgi-bin/blast-fungal.pl was used to survey the *Saccharomyces cerevisiae *genome and those of a number of other wild yeast species (*Saccharomyces bayanus, Saccharomyces castellii, Saccharomyces kluyveri, Saccharomyces kudriavzevii, Saccharomyces mikatae, Saccharomyces paradoxicus, Saccharomyces servizzii, Saccharomyces unisporus, Ashbyagossypii, Candida glabrata, Candida parapsilopsis, Candida tropicalis, Kluveromyces delphensis, Kluveromyces lactis, Kluveromyces marxianus, Kluveromyces thermotolerans, Kluveromyces waltii, Lodderomyces elongisporus*, and *Yarrowia lypolitica*).

All hits with an e value less than e^-10 ^were extracted and an initial phylogenetic analysis used to identify a putative set of ferrichrome NRPSs. The individual A domains of all candidate ferrichrome synthetase NRPSs were aligned with Tcoffee and a phylogeny constructed using the WAG model plus gamma with 100 bootstrap replicates in PhyML [21]. A domains of 12 additional NRPSs found in *C. heterostrophus*, representative of the diverse clades of fungal NRPSs [3], as well as the top bacterial hit (NCBI Accession YP_049592) to both NPS2 and SidC, were used as outgroups in this initial analysis and in further analyses of the complete dataset [3]. A monophyletic clade with bootstrap support > 85% containing all known ferrichrome synthetase NRPSs was identified and all members of this clade were considered in further analyses (see Additional file 1). Two additional known ferrichrome siderophores, one from *Aureobasidium pullulans *(AAD00581) [6] and one from *Omphalotus olearius *(fso1, AAX49356) [22] were included. Several NRPSs identified previously as putative siderophore metabolite producers (designated the SidE clade) [23], which fell in a clade just outside the major clade of known ferrichrome synthetases, were also included.

### Annotation of candidate ferrichrome synthetases

Candidate ferrichrome synthetases were annotated by 1) using the candidate NRPS proteins as queries against the PFAM database and 2) utilizing NRPS specific HMM models built using HMMER [24] from a larger dataset of fungal NRPS A and C domains (KE Bushley and BG Turgeon, manuscript in preparation). Discrepancies between the two methods and with published domain architectures were resolved by manual inspection and adjustment. Individual A domains were extracted using a customized Perl script (available upon request) and the limits of the A domain were defined as in Lee et al [3], spanning from ~33 residues upstream of the A1 core motif to three residues downstream of the A10 core motif [12].

Several proteins identified appeared to be incomplete or incorrectly annotated in the databases. The gene corresponding to *B. cinerea *BC1G15494 (see Additional file 1) is on the end of supercontig 180; we assumed it is incomplete, as it encodes only a single A-T-C module. We reannotated the genes corresponding to HCAG07428 and HCAG07429 as a single gene. The sequence corresponding to *H. capsulatum *HCAG07428 spanning the first C and second A domains is of low quality; the second A domain and the second and sixth C domains are missing from our analyses. Similarly, *U. reesii *UREG00890 and UREG00891 appear to correspond to a single gene. *C. cinerea *CC1G04210 is unusual in that it contains only a single A-T-C module followed by a T-C repeat. Inspection of sequences flanking this gene did not reveal additional A, T, or C domains.

### Phylogenetic analyses

#### Complete set of A domains

A domain protein sequences were aligned to the crystal structure of the A domain of Gramicidin synthetase (GrsA) [25] using 3D-Coffee with the Blosum 62 substitution matrix and default gap opening and extension parameters [26]. Because the alignment of these highly divergent proteins contained regions of ambiguous alignment, we performed a sensitivity analysis to assess the effect of the alignment on the final phylogeny obtained. Starting with the final manually adjusted alignment of A domains, we created and analyzed three different alignments, using maximum likelihood (ML): 1) an alignment retaining the majority of divergent regions, 2) a semi-conservative alignment omitting the most divergent regions (i.e., those with more than 70% gaps per column in the alignment), and 3) a highly conservative alignment with all divergent regions with more than 50% gaps per column removed. The WAG substitution matrix with rate variation described by a gamma distribution with 4 rate categories was identified as the best protein substitution model for this dataset according to the AIC criterion using Protest [27]. ML analyses using the WAG model plus gamma in PhyML showed that the three alignments produced identical topologies for the major clades with only slight differences in groupings of taxa within each clade (available upon request). We used the semi conservative alignment for all further analyses. Phylogenetic analyses were conducted with PhyML using the WAG amino acid substitution model and gamma distribution with 4 rate categories and estimated alpha parameter and 500 bootstrap replicates [21] and with Mr. Bayes using 5 million MCMC generations sampled every 100 generations with a mixed AA prior [28].

The program Genetree [29] was used to reconcile the ML tree to a species tree (see Additionals file 2 and 6) to infer a history of A domain duplications using both duplication and loss as the optimality criterion. The species tree was based on three recent phylogenetic studies of the fungal kingdom [30-32]. These studies agree on placement of all taxa included in this study except the Dothideomycetes whose placement remains unstable. In different types of analyses they have grouped with Eurotiomycetes [31], as more closely related to Sordariomycetes and Leotiomycetes [31], or as basal to all three of these classes [30,31]. We chose to place the Dothideomycetes as sister to other filamentous ascomycetes in the subphylum Pezizomycotina as they are placed in this position in phylogenies with larger taxon sampling [30] and this placement agrees with another recent phylogenomic study [33] (see Additional file 2 and 6). *A. pullulans *was shown to have diverged earlier than our other sampled Dothideomycete taxa in a recent class wide phylogeny of Dothideomycetes and is thus placed at the base of the Dothideomycete clade [34].

#### Individual lineage analyses

To analyze mechanisms of evolution of the genes encoding ferrichrome synthetase proteins in more detail, those enzymes grouping with *C. heterostrophus *NPS2 and those grouping with *A. nidulans *SidC in phylogenetic analyses of the complete A domain dataset (see above) were examined separately. For each group, A and C domains were extracted using the Perl script described above. T domains were excluded, as they are significantly shorter (66 amino acids versus 300 amino acids) and resulted in highly unresolved phylogenies. The limits of the A domain were defined as described above while the C domain was delimited according to the PFAM model (PFAM00668)http://www.sanger.ac.uk/Software/Pfam/ and extends from four residues before the C1 motif to four residues after the C5 motif. Each domain was aligned separately with TCOFFEE using default parameters and phylogenetic analyses were conducted with PhyML and Mr. Bayes using the same parameters described above for the larger dataset. We used A and C domains from the first complete A-T-C module of the SidE group as an outgroup as this module grouped directly outside the major clade of ferrichrome synthetases in both the ML and Bayesian trees while the second module grouped consistently with other types of fungal NRPSs represented by the other *C. heterostrophus *NRPSs.

As the majority of NRPS genes are multimodular, tandem duplication represents a plausible hypothesis for the generation of a multimodular gene from a single A-T-C unit. To evaluate this hypothesis, we constructed phylogenies in PhyML of a representative ferrichrome synthetase from each lineage, i.e., *C. heterostrophus *NPS2 and *A. nidulans *SidC for the NPS2 and NPS1/SidC lineages, respectively. These trees were evaluated using the Possible Duplication History (PDH) algorithm developed to determine if a phylogeny is consistent with a history of tandem duplication [35].

### Substrate specificity

#### Structural modeling

Three-dimensional models of A domains were generated by using template-based modeling techniques. Blast searches [36,37] of the Protein Data Bank (PDB) database [38]http://www.rcsb.org/pdb/home/home.do, using a subset of A domain sequences from *C. heterostrophus *NPS2 (AAX09984), *F. graminearum *NPS2 (FG05372), *F. graminearum *NPS1 (FG11026), *A. nidulans *SidC (AN0607), *U. maydis *sid2 (UM05165), *U. maydis *fer3 (UM01434), and *S. pombe *Sib1 (CAB72227) as queries, indicated a high level of similarity with the phenylalanine activating A domain of the NRPS for gramicidin (GrsA), PDB code: 1AMU; [25]. Using the Combinatorial Extension method [39] and the 1AMU_A (ie., monomer A of 1AMU) structure as input, other structurally similar proteins with associated crystal structures were identified. The structures of the monomers of 1AMU_A, 1PG3_A, 1ULT_A, 1LC_I, 1T5D_X and 1MD9_A were superimposed and a structural alignment of these was produced manually with the help of graphic tools included in the commercial programs ICM (MOLSOFT Inc) and DS-Modeling (Accelrys Inc.). The objective of having a structural alignment of multiple proteins is to better define the regions of the fold that are conserved and understand where structural variability can occur.

The subset of our NRPS A domain sequences (described above) were selected for structural modeling and added to the structural alignment. The alignment was corrected manually by adjusting the positions of insertions and deletions that were incompatible with the secondary-structure elements observed in the 3-dimensional (3D) structures of the templates. All residues forming the walls of the binding pocket for the Phe substrate in 1AMU_A as well as residues that bind the adenosine monophosphate AMP moiety were identified. In addition, residues aligned with the 10 amino acid positions (10AA code) predicted to be involved in substrate specificity in the GrsA sequence [9], as well as three additional residues identified by Schwecke et al. [6] to be important in binding the AHO substrate (13AA code) were identified in the structural alignment. The Cartesian coordinates of the template structures were retrieved from the PDB [38], and the final multiple alignment of the experimental and template structures were used as input data for MODELLER [40-43]. During the process of model generation, MODELLER minimizes the violations of distance and dihedral-angle restraints derived from the templates. For each sequence a set of 3D models were generated and those that best satisfied the set of restraints were kept. More than one template structure was used during the model generation process in order to assess the variability of the different regions of the A domain structures.

#### Evolutionary approaches to identify specificity residues

We utilized several amino acid based methods to detect residues with a potential role in specificity. These included the specificity-determining positions (SDPpred) algorithm [44] and server http://math.genebee.msu.ru/~psn/ and Type I and Type II functional divergence, two likelihood based methods in the DIVERGE 2 package to detect functional residues [45,46]. Type I functional divergence detects changes in evolutionary rates between clusters indicative of changes in constraint or selective pressure, while both the SDP algorithm and Type II functional divergence aim to identify residues that are conserved within a cluster but show a change in amino acid properties between clusters. For these analyses, we used the major groups identified in our ML analysis of all A domains as individual clusters. The second A domain of *S. pombe *sib1 and the third A domain of *O. olearius *fso1 were omitted because both are highly divergent from other A domains and likely degenerate as they lack several core functional motifs [6,9]. The Dothideomycete module 3 A domain was grouped with the cluster for the second *A. nidulans *SidC A domain, as all methods used require clusters of greater than three taxa and our data suggested that all of these domains code for the same amino acid.

## Results

### Distribution of ferrichrome synthetases in fungi

With the exception of *S. pombe *none of the yeast, chytrid, or zygomycete genomes surveyed contained a candidate ferrichrome synthetase NRPS. In contrast, all filamentous ascomycete genomes queried contained at least one and many had two (Table 1). *B. cinerea *appears to have three. For the five basidiomycete genomes examined, two known NRPSs (sid2 and fer3) were found in *U. maydis*, one undescribed ferrichrome synthetase was identified in *C. cinerea *while *P. chrysosporium, L. bicolor*, and *C. neoformans *lacked genes encoding these enzymes. As noted earlier, the ferrichrome synthetase fso1 is known from the basidiomycete *O. olearius *[22].

**Table 1:** Fungal genomes and number of ferrichrome synthetases identified

Species	Number of Ferrichrome NRPSs	Species	Number of Ferrichrome NRPSs
**Hemiascomycetes**		**Ascomycetes**	

*Ashbya gossypii*	0	*Alternaria brassicicola*	1
*Candida albicans*	0	*Aspergillus fumigatus*	1
*Candida glabrata*	0	*Aspergillus nidulans*	1
*Candida parapsilopsis*	0	*Aspergillus niger*	1
*Candida tropicalis*	0	*Aspergillus oryzae*	1
*Kluveromyces delphensis*	0	*Aspergillus terreus*	1
*Kluveromyces lactis*	0	*Botrytis cinerea*	3^a^
*Kluveromyces marxianus*	0	*Chaetomium globosum*	2
*Kluveromyces thermotolerans*	0	*Coccidioides immitis*	1
*Kluveromyces waltii*	0	*Fusarium graminearum*	2
*Lodderomyces elongisporus*	0	*Histoplasma capsulatum*	1^a,b^
*Saccharomyces bayanus*	0	*Magnaporthe grisea*	1
*Saccharomyces castelli*	0	*Neurospora crassa*	1
*Saccharomyces cerevisiae*	0	*Sclerotinia sclerotiorum*	2
*Saccharomyces kluyveri*	0	*Stagonospora nodorum*	1
*Saccharomyces kudriavzevii*	0	*Trichoderma reesii*	1
*Saccharomyces mikatae*	0	*Uncinocarpus reesii*	1^b^
*Saccharomyces paradoxus*	0		
*Saccharomyces servazzii*	0	**Schizosaccharomycetes**	
*Saccharomyces unisporus*	0	*Schizosaccharomyces pombe*	1
*Yarrowia lypolitica*	0		
		**Basidiomycetes**	
**Chytridiomycota**		*Coprinus cinerea*	1
*Batrachochytrium dendrobatis*	0	*Cryptococcus neoformans*	0
		*Laccaria bicolor*	0
**Zygomycota**		*Phanaerochaete chrysoporium*	0
*Phycomyces blakesleeanus*	0	*Ustilago maydis*	2
*Rhizopus oryzae*	0		

^a ^The genes, BC1G15494 and HCAG07428/HCAG07429 are partial (see text).

^b ^HCAG07428 and HCAG07429 and UREG00890 and UREG00891 reannotated as single genes.

### Domain architecture of ferrichrome synthetases

**Figure 2:**
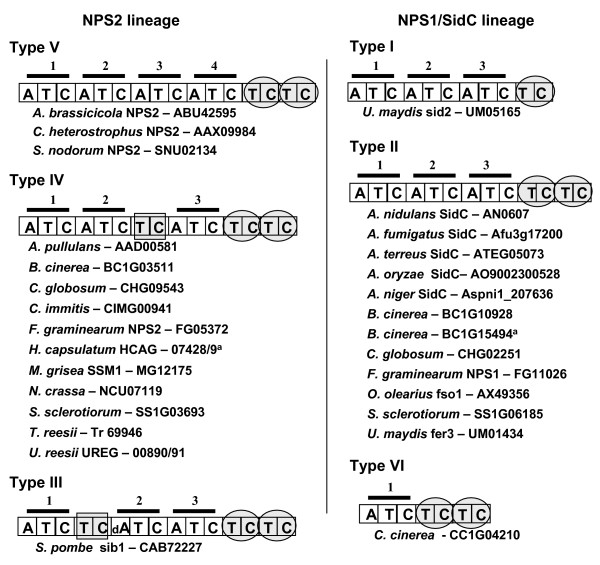
**Six modular architectures for ferrichrome synthetase NRPSs**. Types III, IV, and V are in the NPS2 lineage while Types I, II, and VI are in the NPS1/SidC lineage. A: adenylation domain, T; thiolation domain, C; condensation domain. dA; degenerate A domain. Bars above boxes indicate complete modules. Circles indicate incomplete modules and/or a T-C unit. Superscript 'a' indicates partial gene.

Ferrichrome NRPSs show a diversity of domain architectures (Fig. 2). These have been designated 'types' [6] and we use this terminology here. We found six types, including five previously identified. All are modular (except Type VI), consisting of three to four complete A-T-C modules usually followed by a T-C repeat. *C. heterostrophus *NPS2, as described previously [3,20], has four complete A-T-C modules and a terminal T-C repeat (Type V). This structure is conserved in NPS2 homologs from the other Dothideomycetes examined (*A. brassicicola *and *S. nodorum*). In contrast, most other ferrichrome synthetases examined (Types I – IV) have only three complete A-T-C modules and a terminal T-C repeat. *U. maydis *sid2 (Type I) is an exception, with a single terminal T-C unit. *S. pombe *sib1 (Type III) is the only representative of its class; the second complete modulehas a degenerate A domain in which many of the signature motifs are missing [6] and an internal T-C unit after the first complete A-T-C module. Similarly, all Type IV NPS2 homologs (e.g., *F. graminearum *NPS2) have an internal T-C after the second complete A-T-C module. The only representative of Type VI, *C. cinerea *CC1G04210), has a singleA-T-C module followed by a T-C repeat.

SidE proteins, suggested by Cramer et al [23] to be putative ferrichrome synthetases have a different domain organization from known ferrichrome synthetases. They consist of only two complete modules and an additional N-terminal C domain (5'C-A-T-C-A-T-C3'), except for *A. fumigatus *Afu3g03350 and Afu3g15270 which lack the N-terminal C domain(5'A-T-C-A-T-C3').

Thus, although at least one representative of each Type (except Type VI) has been shown to produce the conserved ferrichrome siderophore compound consisting of six substrates (three amino acids and three AHO units) (Fig. 1), the domain architectures of the ferrichrome synthetases responsible for their biosynthesis vary considerably.

### Two distinct lineages of ferrichrome synthetases

**Figure 3:**
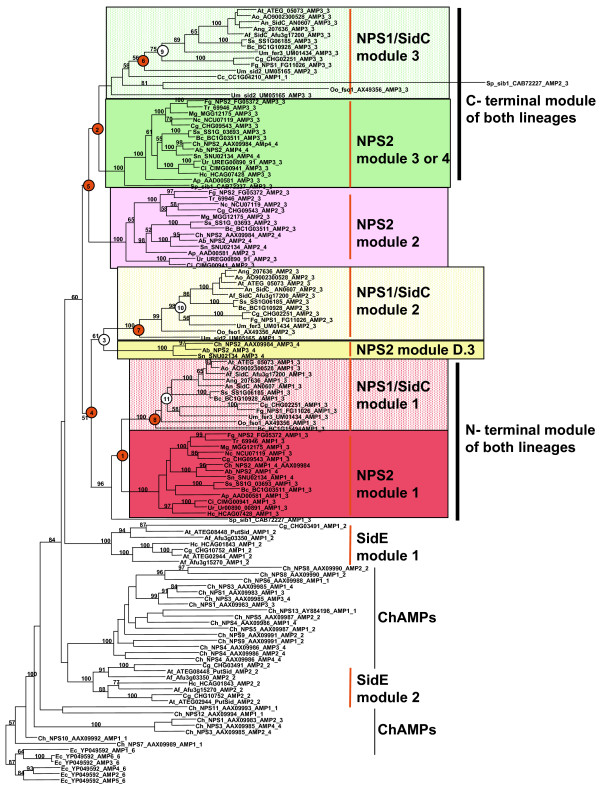
**Maximum likelihood tree of all AMP domains examined in this study demonstrating two separate lineages of ferrichrome synthase NRPSs**. N-terminal A domains of both lineages group together and C-terminal domains of both lineages group together (thick vertical bars). NPS2, module 2 groups with the C-terminal modules, while NPS1/SidC module 2 and Dothideomycete NPS2 module D.3 group with the N-terminal modules. Numbered nodes indicate duplications inferred from the reconciliation analysis. White circles indicate a duplication inferred due to incongruence of the gene tree with the species tree (see Additional files 2 and 6), while red circles indicate a duplication inferred due to the presence of two copies of a gene in the same species. Bootstrap support values greater than 50% are reported above branches. Note that the A domains of SidE module 1 group as directly sister to all ferrichrome synthetase A domains examined here, while A domains of SidE module 2 group with A domains of other types of *C. heterostrophus *NRPSs. For species and protein Accession numbers see Additional file 1. Nomenclature: e.g., Ch_ NPS2_AAX09984_AMP3_4 indicates *C. heterostrophus*, protein accession number AAX09984, AMP module 3 of a total of 4 (see Fig. 2). For Bayesian analysis, see Additional files 3 and 6.

Both methods of phylogenetic analysis of A domains from the complete dataset showed a history of domain duplications that supports the hypothesis of at least two separate lineages of fungal ferrichrome synthetases (Fig. 3, see Additional files 3 and 6). For all A domains, we find two clades whose members correspond to homologs of *C. heterostrophus *NPS2 or to *A. nidulans *SidC. For convenience, we call the lineage represented by *C. heterostrophus *and *F. graminearum *NPS2 (Types V and IV, respectively, Fig. 2), the NPS2 lineage. The other lineage, represented by *A. nidulans *SidC, *U. maydis *fer3, *F. graminearum *NPS1, *U. maydis *sid2 and *C. cinerea *CC1G04120 (Types I, II and VI, Fig. 2), we call the NPS1/SidC lineage. Some species, e.g., *F. graminearum, B. cinerea, C. globosum, S. sclerotiorum *have representatives in both lineages. Others, such has *U. maydis *and *B. cinerea*, have more than one representative within the NPS1/SidC lineage.

The reconciliation analysis clearly identified duplication nodes giving rise to the first (N-terminal, node 1, red boxes) and final (third or fourth) (C-terminal, node 2, green boxes) A domains of both lineages (Fig. 3). This analysis also provides support for a relationship at node 3 between the third A domain of NPS2 Type V of the Dothideomycetes (D.3) and the second A domains of NPS1/SidC Type II (Fig. 3, yellow boxes).

ML and Bayesian phylogenetic methods support the duplication at node 1, giving rise to the N-terminal A domains of both lineages (red boxes), with high Bayesian posterior probability (pp = 1.00) but low ML bootstrap support (bs < 50%) (Fig. 3, see Additional files 3 and 6). The duplication at node 2, giving rise to the C-terminal A domains of members of both lineages (Fig. 3, green boxes), is weakly supported by both types of phylogenetic analysis (bs < 50%), pp = .74) (Fig. 3, see Additional files 3 and 6). For the internal modules, both ML and Bayesian analyses group the third

A domain (D.3) of NPS2 Type V and the second A domain of the NPS1/SidC lineage together (yellow boxes), supporting a duplication at node 3 inferred by the reconciliation analysis (Fig. 3, see Additional files 3 and 6). The Bayesian analysis provides higher support (pp = 1.00) for this relationship than does the ML analysis (bs = 61%). These clades (yellow boxes) group with the N-terminal modules of both lineages (Fig. 3, red boxes), with higher Bayesian (pp = 1.00) than ML (bs = 51%) support; a duplication at node 4 was inferred by the reconciliation analysis. Finally, the module 2 A domains of NPS2 Types IV and V (pink boxes) group together and with the C-terminal modules of both lineages (Fig. 3, green boxes), however with weak support (bs < 50% and pp = .74). The reconciliation analysis identified a duplication at node 5 corresponding to this relationship (Fig. 3).

**Figure 4:**
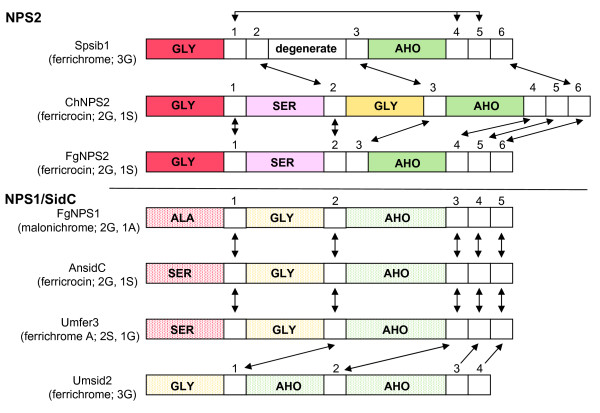
**Schematic representation of phylogenetic relationships among A and among C domains within each lineage**. A domain relationships for each lineage and between lineages are color coded as in Fig. 3 and Additional files 3 and 6. C domain relationships are indicated by arrows for each lineage. The NPS2 lineage relationships are indicated in the top half of figure and the NPS1/SidC lineage relationships in the bottom half of figure. Scheme is based on phylogenetic analyses of A (Fig. 3, see Additional files 3, 4 and 6and C domains. Spsib1, ChNPS2, FgNPS2, FgNPS1, AnSidC, Umfer3, and Umsid2 are representative of architectural Types I-V (Fig. 2). Also mapped on the A domains are predicted substrates adenylated by each domain, based on structural modeling (Table 2, Fig. 7). SER = serine, GLY = glycine, ALA = alanine, AHO = N^5^-acyl-N^5^-hydroxy-L-ornithine. Within the NPS2 lineage, ChNPS2 and FgNPS2 C domain analyses clearly indicate that C2 domains are related, as are C3 domains. Thus the difference in protein architecture in this region is presence/absence of an A domain between C2 and C3. A similar argument can be made for the difference in protein structure between C1 and C2 C domains of Spsib1 *vs *those of ChNPS2 and FgNPS2. For the NPS1/SidC lineage, A and C domain analyses of FgNPS1, AnSidC, and Umfer3 clearly indicate that there is a one to one relationship for all A and all C domains. Examination of Umsid2, however, indicates that Umsid2 module 1 A domain is related to the module 2 A domains of the other members of this group, while Umsid2 modules 2 and 3 A domains are related to the C-terminal module of the other members of this group. Umsid2 appears to lack the N-terminal A domain of other NPS1/SidC members, since the C domain from module 1 is related to the C domains of module 2 of the rest of the lineage. Similarly the C domains from Umsid2 module 2, 3, 4 are related to the C domains of modules 3, 4, 5 of the rest of the NPS1/SidC lineage.

The phylogenetic relationships of A domains are mapped by color to representative ferrichrome synthetases in Fig. 4 (color corresponds to clades identified in Fig. 3). These data clearly show that the N-terminal and C-terminal A domains of each lineage are related by duplication (Fig. 4). Similarly, the third A domain of the Dothideomycete Type V (D.3) proteins appears related to the second A domain of the NPS1/SidC lineage by duplication (yellow). The second module of Dothideomycete Type V, which is the only type of ferrichrome synthetase consisting of four complete A-T-C modules (Fig. 2), does not have an obvious counterpart in other ferrichrome synthetases (Fig. 4, pink).

### Additional duplications within the NPS1/SidC lineage

There is evidence for further duplications within the NPS1/SidC lineage. The reconciliation analysis identified duplication nodes at 6, 7, and 8 (Fig. 3) due to the presence of two representatives from the NPS1/SidC lineage in both *U. maydis *[UM01434/fer3 (Type II) and sid2 (Type I)] and *B. cinerea *[BC1G10928 and BC1G15494 (Type II)] (Fig. 2). Duplication nodes were also identified due to the incongruence of *F. graminearum *FG11026 (NPS1) and *C. cinerea *CHGG02251 with the species phylogeny at nodes 9, 10, and 11 where these two NRPSs group with or outside of basidiomycete *U. maydis *fer3 rather than with other ascomycete NRPSs (Fig. 3). Thus, the data provide support for one and possibly two additional bifurcations within the NPS1/SidC lineage.

The placement of certain NPS1/SidC lineage genes is ambiguous. Type VI *C. cinerea *CC1G04210 has a single A domain which groups consistently with the third A domain of *U. maydis *sid2 (Fig. 3, see Additional files 3 and 6). The other basidiomycete gene, *O. olearius *fso1, tends to group with other Type II NPS1/SidC proteins. In both analyses, the first and second modules of fso1 group at the base of the clades containing the corresponding modules of the NPS1/SidC Type II proteins, usually with *U. maydis *fer3 (Fig. 3, see Additional files 3 and 6). The third fso1 A domain is highly diverged and contains degenerate core motifs and its placement varies (Fig 3, see Additional files 3 and 6). The single A-domain of incomplete *B. cinerea *BC1G15494 tends to group at the base of the clade containing the first A domain of all Type II NPS1/SidC proteins (Fig. 3, see Additional file 3 and 6), however, in both ML and Bayesian analyses (Fig. 3, see Additional files 3 and 6), it shows incongruence with the species phylogeny by grouping outside of basidiomycete NRPSs in this clade.

### S. pombe *sib1*

The relationship of Type III *S. pombe *sib1 to other ferrichrome synthetases is ambiguous. In both the ML and Bayesian analyses, the first A domain of sib1 groups as sister to the first A domains of both the NPS2 and NPS1/SidC lineages (Fig. 3, see Additional files 3 and 6) with fairly high support (bs = 96% and .89 pp), suggesting an ancestral relationship of this sib1 A domain and the first A domains of both lineages. However, the sib1 module 3 A domain groups with the A domains of NPS2 terminal modules 3 or 4, in both trees (Fig. 3, see Additional files 3 and 6), with strong support (bs = 100% and pp = 1.00). The sib1 module 2 A domain groups with the module 3 A domain of the NPS2 lineage (Type V) with high support in the Bayesian analysis (pp = 1.00) (see Additional files 3 and 6). In the ML tree, however, it groups with the N-terminal A domain of the NPS1/SidC lineage (Fig. 3), but without bootstrap support. As discussed above, this second A domain is highly diverged, lacks several core A domain motifs [9], and as suggested by Schwecke [6], is likely nonfunctional. As sib1 most consistently groups with homologs of *C. heterostrophus *NPS2, we placed it in the NPS2 lineage (Fig. 2).

### Putative ferrichrome synthetases in the sidE clade

The SidE proteins, identified as putative ferrichrome synthetases [23], group as sister to all other known ferrichrome synthetases (Fig. 3, see Additional files 3 and 6). The A domains of the first and second modules of these proteins however, are not monophyletic. In the ML and Bayesian analyses, SidE module one A domain groups as sister to known ferrichrome synthetases while the SidE module two A domain groups with other (non-ferrichrome synthetase) NRPSs from *C. heterostrophus*. Thus, these results suggest that only the first module of the SidE proteins is clearly related to other known ferrichrome siderophore NRPSs.

### Individual lineage analysis

**Figure 5:**
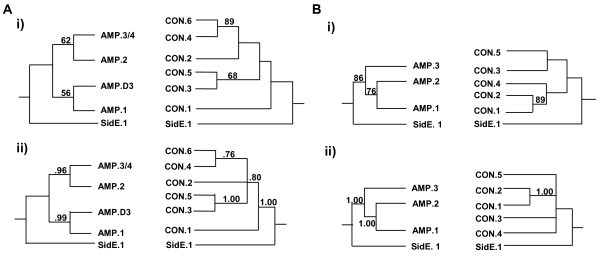
**Diagrammatic depiction of separate NPS2 (A) and NPS1/SidC (B) lineage AMP and CON domain trees. (i) and (ii) are ML and Bayesian analyses, respectively**. A. Relationships among A and among C domains in the NPS2 lineage. As demonstrated in the full A domain dataset analyses (Fig. 3, see Additional files 3 and 6), both NPS2 lineage A analyses support a relationship between C-terminal modules 3 or 4 and module 2, and a relationship between N-terminal module 1 and Dothideomycete module D.3. For the C trees, both analyses support a relationship between C4, and C6 (bs = 89% and pp = .76) and between C3 and C5 (bs = 68% and pp = 1.00). C2 groups with C4 and 6 in the ML analysis and with C3-6 in the Bayesian analysis but without support in either case. In both trees, C1 is ancestral, but without support. B. Relationships among A and among C domains in the NPS1/SidC lineage. As demonstrated in the full A domain analyses (Fig. 3, see Additional files 3 and 6), both NPS1/SidC lineage A domain analyses support a relationship between N-terminal module 1 and module 2, and indicate C-terminal module 3 is ancestral. Similarly, the ML and Bayesian trees support a close relationship between the C domains of modules 1 and 2.

The backbones of the A and C tree topologies for each lineage, rooted with the first module of the SidE clade, are shown in Figs. 5A and 5B. Within each lineage, all A and all C domains fall into well-supported monophyletic clades (see Additional files 4A–D and 6). A domain relationships are consistent with those of the full A dataset (compare Additional file 4A with Fig. 3). The first through the sixth C domain of all proteins group together as separate clades for all members of the NPS2 (except *S. pombe *sib1) and the NPS1/SidC lineages (Fig. 5, see Additional files 4B, 4D and 6). C domain relationships among representative ferrichrome synthetases are shown in Fig. 4 (arrows).

For the NPS2 lineage (Fig. 5A), both A domain tree topologies (ML and Bayesian) support a close relationship between module one A domains of all types (I, IV, V) and the A domain of Dothideomycete Type V module 3 (D.3) (bs = 56% and pp = .99) (Fig. 5A, see Additional files 4Ai–ii and 6). A close relationship is also supported between module 2 A domains of Types IV and V and the terminal module A domains of all types (bs = 62%, and pp = .96) (Fig. 5A, see Additional files 4Ai–ii and 6). The ML and Bayesian analysis of the C domains (Fig. 5A, see Additional file 4Bi–ii and 6) support a close relationship between modules 4 and 6 C domains and between module 3 and 5 C domains (bs = 89% and pp = 0.76, bs = 68% and pp = 1.00, respectively).

**Figure 6:**
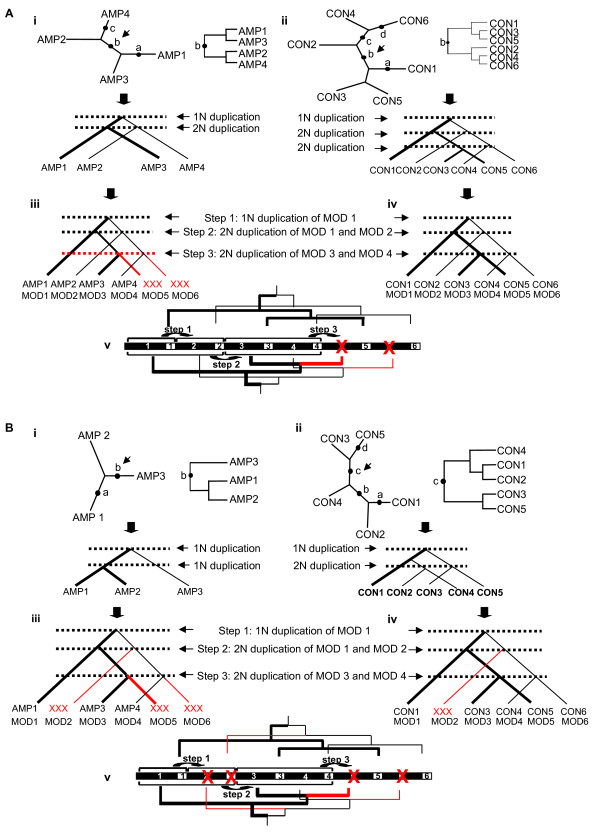
**Evaluation of *C. heterostrophus *NPS2 and *A. nidulans *SidC with the PDH algorithm (possible duplication history)**. A. i) Unrooted maximum likelihood phylogeny of *C. heterostrophus *NPS2 A domains, the duplication tree resulting from rooting the phylogeny at position b (top) and inferred partially ordered duplication history (below). ii) Unrooted maximum likelihood phylogeny of *C. heterostrophus *NPS2 C domains, the duplication tree resulting from rooting the phylogeny at position c, and partially ordered duplication history (bottom). iii) and iv) Representation of the series of three tandem duplication events suggested by the partially ordered duplication trees of C domains. Bold and thin lines indicate relationships among modules 1, 3, and 5 and among modules 2, 4 and 6 respectively. If one infers loss of AMP5 and AMP6, relationships among A domains are consistent with the series of three tandem duplication events inferred from the C domain partially ordered duplication history: Step 1) duplication of A module 1, Step 2) duplication of A modules 1 and 2, and Step 3) duplication of A modules 3 and 4. v) Relationships among A and among C domains in partially ordered duplication histories mapped to the domain architecture with predicted domain losses shown in red. B. i) Unrooted maximum likelihood phylogeny of *A. nidulans *SidC A domains, duplication tree rooted at position b (top) and inferred partially ordered duplication history (bottom). ii) Unrooted maximum likelihood phylogeny of *A. nidulans *SidC C domains, duplication tree rooted at position c (top) and inferred partially ordered duplication history (bottom). iii) and iv) Representation of the series of three tandem duplication events suggested by the partially ordered duplication trees. Bold and thin lines as in A above. Relationships among *A. nidulans *SidC A domains are consistent with the series of tandem duplication events predicted by relationships among the *C. heterostrophus *NPS2 C domains if losses of AMP2, AMP5, and AMP6 are invoked (iii). Relationships among SidC C domains are also consistent with a series of three tandem duplication events if loss of CON2 is invoked (iv). v) Relationships from partially ordered duplication histories mapped to the domain architecture with predicted domain losses shown in red.

The unrooted ML phylogenies of the A and C domains of *C. heterostrophus *NPS2 are shown in Figs. 6 Ai and Aii. When the C tree is rooted at position b (Fig. 6Aii) and evaluated with the PDH algorithm [35], the resulting phylogeny is a duplication tree that implies an associated partially ordered duplication history (Fig. 6Aii). All trees with four taxa are true duplication trees, thus evaluation of the A domains with the PDH algorithm is trivial. However, the duplication tree resulting from rooting the A domain phylogeny at b implies a partially ordered duplication history which also infers a duplication between modules 1 and 3 and between modules 2 and 4, consistent with duplications predicted for C domains (Figs. 6Ai, ii).

For the NPS1/SidC lineage, the A domain phylogenies show a strong relationship between A domains of modules 1 and 2 (bs = 76% and pp = 1.0) (Fig. 5B, see Additional files 4Ci–ii and 6). Both the ML and Bayesian trees for the C domains also support a strong relationship between modules 1 and 2 (Fig. 5B, see Additional files 4Di–ii and 6). The ML tree also groups C domains 1, 2 and 4 together and C domains 3 and 5 together, although there is poor bootstrap support for these relationships. The Bayesian tree was unresolved with respect to the remaining C domains. The relationships of A domains in the phylogeny of the complete dataset (Fig. 3, see Additional files 3 and 6) suggest that the second A domain of the NPS1/SidC lineage corresponds to the third A domain (D.3) of the NPS2 lineage (Figs. 2, 3 and 5). Thus, the NPS1/SidC lineage analyses also support a relationship between A domains corresponding to the first and third modules of the NPS2 lineage.

The unrooted ML phylogenies of A and C domains from *A. nidulans *SidC are shown in Fig. 6Bi, Bii. When the tree of SidC C domains is rooted at position c (Fig. 6Bii), and evaluated with the PDH algorithm [35], the resulting tree is a duplication tree which implies the partially ordered duplication history shown in Fig. 6Bii. Similarly, the SidC A domains are duplication trees with an associated partially ordered duplication history (Fig. 6Bi) that is also consistent with the duplication history predicted for SidC domains.

### Adenylation domain substrate choice

#### Structural modeling

**Figure 7:**
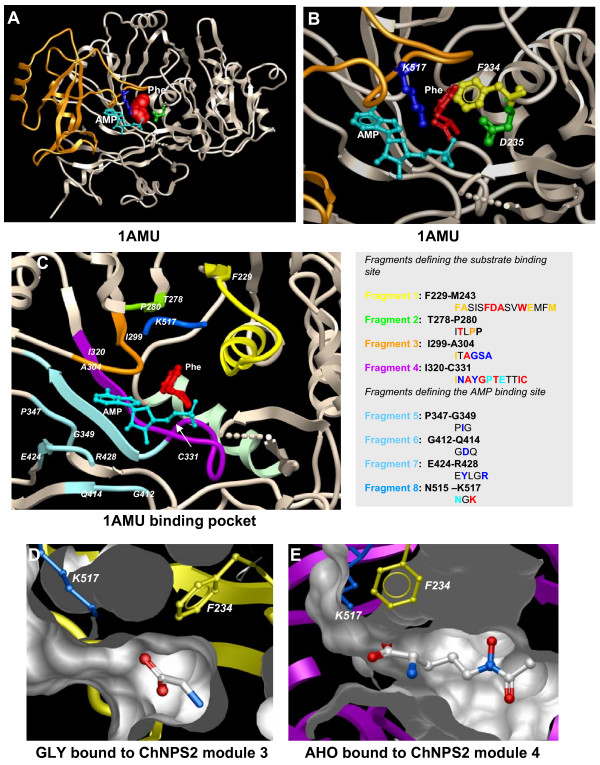
**3D modeling of selected NRPS AMP binding domains**. A. Ribbon representation of the structure of the activated domain of Gramicidin synthetase (PDB code: 1AMU) bound to its Phe substrate (shown as a CPK model; red) and adenosine monophosphate (AMP; shown as "*ball & stick*" representation of the heavy atoms; light-blue). The large domain (gray ribbon), contains the substrate and AMP binding pockets. A second smaller domain (orange), involving residues D430 to F530, sits at the entrance of these pockets. "*Ball & stick*" representations of residues D235 and K517 are shown in green and blue, respectively. B. View of the GrsA binding pockets for Phe and AMP showing the positions of the conserved residues F234 (yellow), D235 (green), and K517 (blue). D235 and K517 are in contact with the amino and carboxyl end groups, respectively, of the Phe substrate. C. Alternative view of GrsA highlighting all the fragments of the sequence that determine the binding pockets for Phe and AMP. The amino acid composition of those fragments is listed to the right. The color convention for the residues is as follows: red and orange indicate those residues lining the substrate cavity, with residues in red making contact with the substrate Phe in the experimental structure; blue and light blue indicate residues lining the AMP binding site, with residues in blue making contact with AMP in the experimental structure. D. Slice through the substrate binding site of a 3D model of ChNPS2 module 3. The central cavity is packed with large residues that produce a shallow pocket. A *ball & stick *representation of a bound GLY residue is also shown to help assess the size of the cavity (compare to Fig. 7E). E. Slice through the substrate binding site of a 3D model of ChNPS2 module 4. The central cavity is lined with small residues that leading to a deep pocket. A *ball & stick *representation of a bound AHO is also shown to help assess the size of the cavity (compare to Fig. 7D).

The experimental structure of Gramicidin GrsA [25] bound to its substrate, phenylalanine (1AMU_A), identified a number of residues that may be relevant for substrate specificity. In the GrsA structure, the binding pocket is formed by residues at the interface between five β-strands (strand 1; D224 to F229, strand 2; T275 to P280, strand 3; Q296 to A301, strand 4; V317 to Y323 and strand 5; A332 to V336) of a β-sheet, two α-helices (helix 1; D203 to S217 and helix 2; D235 to L245) and at some of the loop regions connecting these secondary structure elements (Figs. 7A–C). In addition, a loop (S514 to K517) protruding from a small domain of the protein covers the entrance to the active site region (Figs. 7B–C). A number of sites with the potential to be in direct contact with the substrate, as well as those lining the cavity in such a way that the side chain could affect the size of the binding pocket, were investigated in this work for a possible role in substrate specificity (Table 2). These key residue positions are 229, 230, 240, 243, 280, 320, and 326, plus those in the 10AA 'code' (235, 236, 239, 278, 299, 301, 322, 330, 331, and 517)(Fig. 7C, Table 2). Position 229 was reported previously as part of the 13AA code predicted for the substrate AHO [6], but the additional residues we examined that are not in the 10AA code have not been implicated previously in substrate binding.

**Table 2:** Key positions in AMP domain binding pocket identified by structural modeling

**AMP domain**^a^	**Position**^b^																	Prediction
		
	*2*	2	2	2	2	2	2	2	2	2	3	3	3	3	3	3	5	
	*2*	3	3	3	3	4	4	7	8	9	0	2	2	2	3	3	1	
	*9*	0	5	6	9	0	3	8	0	9	1	0	2	6	0	1	7	
1AMU_A	F	A	**D**	A	W	E	M	T	P	I	A	I	A	T	I	C	**K**	Phe

**Spsib1 AMP1**	F	A	**D**	V	F	E	G	E	T	I	I	V	A	T	I	H	**K**	G
**ChNPS2 AMP1**	F	A	**D**	V	F	E	F	E	T	L	I	W	M	T	I	H	**K**	G
**FgNPS2 AMP1**	F	A	**D**	V	F	E	F	E	T	L	I	W	M	T	I	H	**K**	G

**FgNPS1 AMP2**	L	S	**D**	V	Q	D	Y	H	T	T	I	Y	T	A	V	V	**K**	G
**AnsidC AMP2**	F	S	**D**	V	Q	D	Y	H	T	T	I	F	T	A	V	V	**K**	G
**Umfer3 AMP2**	F	S	**D**	V	Q	D	W	H	T	T	I	Y	T	A	V	V	**K**	G

ChNPS2 AMP3	Y	A	**D**	M	Y	D	L	D	T	Y	I	V	S	T	F	C	**K**	G
Umsid2 AMP1	Y	S	**D**	L	M	D	Y	L	T	I	G	L	L	A	L	I	**K**	G

**ChNPS2 AMP2**	A	C	**D**	V	F	E	F	S	T	V	A	Y	G	S	N	I	**K**	S
**FgNPS2 AMP2**	A	C	**D**	V	F	E	Y	S	T	V	A	W	G	S	N	I	**K**	S

**AnsidC AMP1**	F	A	**D**	P	M	E	V	M	T	W	M	V	A	T	I	N	**K**	S
**Umfer3 AMP1**	F	A	**D**	P	M	E	V	M	T	W	M	A	A	T	V	N	**K**	S

FgNPS1 AMP1	G	A	**D**	I	F	E	W	N	T	M	G	F	G	T	I	Y	**K**	A
Spsib1 AMP2	T	A	**D**	C	C	W	G	I	T	Y	Y	I	A	L	I	C	**K**	degenerate

**Spsib1 AMP3**	F	A	**D**	V	L	E	F	D	T	I	G	Y	F	T	I	G	**K**	AHO
**ChNPS2 AMP4**	F	A	**D**	V	L	E	W	D	T	I	G	Y	G	T	I	G	**K**	AHO
**FgNPS2 AMP3**	F	A	**D**	V	L	E	W	D	T	I	G	Y	A	T	I	G	**K**	AHO

FgNPS1 AMP3	L	T	**D**	P	T	Q	V	G	V	T	G	F	F	T	I	G	**K**	AHO
AnsidC AMP3	Q	A	**D**	P	L	E	F	S	V	T	G	V	A	T	I	G	**K**	AHO
Umfer3 AMP3	L	A	**D**	V	S	Q	M	S	V	G	G	L	A	T	I	M	**K**	AHO
Umsid2 AMP2	R	S	**D**	V	L	E	L	C	V	I	G	L	A	S	I	G	**K**	AHO
Umsid2 AMP3	L	A	**D**	V	I	E	M	D	P	M	G	I	A	T	I	G	**K**	AHO

**a **AMP domains in bold within blocks have highly similar residue sets.

**b **Positions in bold correspond to the proposed 10 AA code. Position 229, in bold italics, corresponds to one of three additional positions (226, 229, 276) predicted by Schwecke et al. [6] to bind AHO. All other sites were identified in this study. Residues D and K at positions 235 and 517 in bold indicate residue conservation. Letters in body of table refer to amino acid residues.

Two sites of key importance for binding amino acid substrates correspond to D235 and K517. In the GrsA structure, the carboxyl group of D235 interacts electrostatically with the amino group of the substrate residue (phenylanalnine), providing one of the anchoring points for the substrate in the binding cavity, while K517 protrudes from a small domain (involving residues D430 to F530) that sits close to both the substrate as well as to the AMP binding pocket (Fig. 7B) [25,9]. Positively charged K517 appears to act as a gatekeeper, lying at the entrance of the active site cavity and projecting its NH_3 _group toward the carboxyl group of the phenylalanine substrate [9,25]. D235 and K517 are conserved across all A domains we examined and thus, though clearly important for substrate binding, should not be considered as residues involved in distinguishing among amino acid substrates (Table 2).

AHO and amino acid substrate assignments for A domains are shown in Table 2 and Fig. 4. A domains of all terminal modules were predicted to code for AHO based on a larger binding pocket size which, in most cases, includes one or two negatively charged residues or a few polar residues (Table 2, Fig. 7E, compare with Fig. 7D). Besides these features, there is no clear pattern based on the residues lining this cavity, except for high similarity among Spsib1, ChNPS2 and FgNPS2 terminal A domain residues (Table 2).

Assignment of the remaining A domains was even more difficult. We found that the consensus 10AA codes for SER, ALA, and ORN identified by Stachelhaus et. al. [9] were not represented in the A domains of ferrichrome synthetases we examined and thus we could not simply infer specificity. Initially, to search for patterns representative of A domains binding SER, ALA, GLY, and ORN, structural alignments of A domains predicted [47,9] to bind these substrates were created (see Additional file 5). The small number of fungal and bacterial domains confirmed to be associated with known substrates makes comparing key fungal positions to the bacterial code positions problematic. We found, however, that bacterial A domain 10AA 'codes' for the same substrate appeared more conserved than fungal ones. The fungal A domains were either too variable or too few for us to deduce a consensus 'code' (see Additional file 5). We did not find any consistent pattern associated with A domains coding for ALA, GLY, or ORN. For SER, however, we found that the majority of sequences share a histidine (HIS) residue at position 278 that our 3D-models suggest is projecting from the top of the binding pocket (Table 2). A domains from FgNPS1, AnsidC, and Umfer3 module 2, have HIS at 278, and their cavities are quite hydrophilic and lined by similar sets of residues (Table 2). We initially considered these modules as the domains most likely to bind SER. We also found that A domains from Spsib1, ChNPS2, and FgNPS2 module 1 share highly similar binding pockets (Table 2), with a HIS at position 331 whose side chain may occupy the center of the cavity (i.e., similar to H278 in our structural alignment) but projecting from the bottom of the pocket), making them, by analogy, also probable candidates to bind SER. The chemistry, however, indicates that Spsib1 produces ferrichrome which contains three glycines and no serine (Fig. 4). Therefore, we infer that the A domain of the Spsib1 module 1 must bind GLY, since it is the only non degenerate A domain, other than the terminal A domain which we predict binds AHO (Figs. 1, 4). Due to the high similarity of the residues forming the AMP cavity of ChNPS2 and FgNPS2 module 1 to those in Spsib1 module 1 (Table 2), we predict these two domains are also likely to bind GLY. By default, module 2 of FgNPS2 is predicted to bind SER (Fig. 4). Based on similarities to the FgNPS2 module 2 binding pocket, ChNPS2 module 2 is predicted to bind SER also (Table 2). Finally, ChNPS2 module 3, which 3D models show has a very crowded and small binding pocket is expected to bind to GLY (Table 2, Fig. 4, Fig. 7D).

AnSidC has been shown to produce ferricrocin [17,48], which contains two glycines and one serine, while FgNPS1 produces malonichrome containing two glycines anda single alanine (G. Adam, BG Turgeon, unpublished) and Umfer3 makes ferrichrome composed of three glycines [7]. As noted in Table 2, key residues in the binding pockets of the second A domains of FgNPS1, AnSidC, and Umfer3 are highly similar to each other and should likely code for a residue that is common between ferricrocin and malonichrome (i.e., GLY). By default, we infer that module 1 of AnSidC and Umfer3 bind SER (Table 2) while module 1 of FgNPS1 binds ALA. 3D modeling shows that the center of these binding pockets are likely filled by many hydrophobic residues. In the case of module 1 of AnSidC and Umfer3, the characteristics of the binding pockets (i.e., highly hydrophobic) do not seem very compatible with binding a hydrophilic residue such as SER. However, an asparagine residue at position 331 in both modules may be able to provide a hydrogen-bond partner to "dock" the side chain of the SER substrate. Lastly, 3D models of Umsid2 module 1, indicate that the binding region must be filled with many hydrophobic residues (Table 2) leading to a very shallow pocket, likely to be selective for GLY.

Thus, we found that the 10AA code failed when we tried to *infer *the specificity of the sequences we examined. Instead, A domains predicted to code for the same substrate [e.g., ChNPS2 AMP1 (GLY) and AnSidC AMP2 (GLY)] had widely divergent 'codes' (Table 2, see Additional file 5) and appeared to diverge according to our A domain phylogeny (e.g., 'codes' for GLY, SER, or ORN are conserved among members of the NPS2 and SidC lineages but differ between the two lineages) (Table 2, Fig. 3, see Additional files 3 and 6). It is noteworthy that, even when protein structural modeling is brought to bear on the issue of key residues 'coding' for substrate specificity, no simple rule was found to be applicable to all sequences considered in this study. While it was possible to infer the size and some properties that characterize the binding pockets, highly divergent residue arrangements appear to bind the same substrate (Table 2, see Additional file 5).

#### Evolutionary approaches to identification of specificity residues

The SDP, Type I and Type II functional divergence analyses identified, with high probability, a number of positions indicating either a shift in amino acid properties between clusters (SDP and Type II) or a shift in evolutionary rate between clusters reflective of changes in evolutionary constraint or selective pressure (Type I) (Table 3). For Type I analyses, all comparisons of paralagous clusters showed θ_I _values significant at p ≤ .05 while for Type II analyses, only comparisons between NPS2 AMP1 and NPS2 AMP 4 (θ_II _= .224 ± .113) and between NPS2 AMP2 and NPS2 AMP4 (θ_II _= .283 ± .113) were significant at p ≤ .05. Several positions received high support from all three methods including positions 252, 278, 301, 322, and 331. Several of the positions identified by structural modeling (230, 239, 243, 278, 299, 301, 320, 322, 326, 330, and 331) also received support from at least one method (Table 2, Table 3). Clusters of significant residues map to the first and second α-helices and to β-strands 2–4, as well as to fragments 1–4 identified by structural modeling as lining the 1AMU_A binding pocket and connecting these key structural features (Table 3; Fig. 7C). Two exceptions to this pattern map to region 246–257 which is on β-strand near the surface of the protein (therefore not located close to the substrate binding site) and region 306–314, which is on a small helix on the surface of the first monomer of 1AMU_A containing both the substrate and AMP-binding pockets. Thus, residues predicted to be involved in functional divergence point to many of the same key regions of the binding pocket predicted by structural modeling to have a potential role in substrate specificity.

**Table 3:** Residues showing evidence of functional divergence in SDP and DIVERGE2 analyses

Position 1AMU_A	Feature	Fragments (Fig. 7)	SDP	Type II	Type I	Position 1AMU_A	Feature	Fragments (Fig. 7)	SDP	Type II	Type I
200				.95		270					.96

201				.78		271					

202						272					

203	**α-Helix 1**				.95	273					
		
204	**D203-S217**			.85	.95	274			5.41		.75
		
205					.90	275	**β-Strand 2**			.95	
				
206			6.56	.95		**276**	**T275-P280**		6.26	.88	
				
207					.80	277			6.44		
				
208				.77	.87	**278**		**x fragment 2**	9.43	.90	.87
					
209						279		**T278-P280**			.97
					
210						280		**x**			
		
211			5.5			281					
		
212					.94	282			7.2		
		
213					.94	283			6.54		.93
		
214			10.7	.95		284					.92
		
215						285					
		
216						286					
		
217						287					

218						288					

219						289			6.68		

220						290					

221						291					

222						292					

223						293					

224	**β-Strand 1**					294					
				
225	**D224-F229**					295					
				
**226**			9.16			296	**β-Strand 3**				.81
				
227						297	**Q296-A301**				.80
				
228						298					.98
				
**229**		**x fragment 1**				**299**		**x fragment 3**	7.88		.75
					
230		**x F229-M243**		.90		300		**I299-A304**			.84
				
231			6.76	.74		**301**		**x**	10.82	.95	.74
				
232			8.92	.75		302				.75	
				
233				.95		303				
				
234						304			5.94		.94
		
**235**	**α-Helix 2**	**x**				305					
			
**236**	**D235-L245**	**x**				306			6.71	.95	
			
237					.90	307					.97
			
238						308				.70	.81
			
**239**		**x**	8.59		.95	309					.77
			
240		**x**				310			7.02		.98
			
241			6.00	.95		311					
			
242						312					.97
			
243		**x**	5.64		.88	313			8.31		
		
244			5.58		.94	314					
		
245						315					

246					.95	316					

247				.87	.99	317	**β-Strand 4**				
		
248					.85	318	**V317-Y323**				
		
249					.98	319					
		
250					.98	320		**x fragment 4**			.78
			
251					.99	321		**I320-C331**	6.46	.76	
			
252			5.99	.85	.93	**322**		**x**	8.25	.90	.90
			
253						323					
		
254				.70	.93	324					
		
255						325					
		
256				.73 C		326		**x**	5.98		
		
257					.97	327				.80	
		
258						328					
		
259			5.47			329				.95	
		
260						**330**		**x**		.95	.71
		
261						**331**		**x**	12.46	.80	.94

262						332	**β-Strand 5**				.90
		
263						333	**A332-V336**				
		
264						334					
		
265						335					

266						336					

267											

268						**517**		**x**			

269											

Left to right columns: 1) positions in 1AMU_A, bold are sites corresponding to the 10 or 13 AA code. 2) Loops and strands in 1AMU_A (Fig. 7A). 3) Fragments defining the substrate binding site; 'x' indicates key sites identified by structural modeling (Fig. 7C, Table 2). 4) Sites identified using the SDP algorithm showing significant Z-scores. 5), 6) Sites identified using tests for Type II and Type I functional divergence, respectively. The highest posterior probability for sites above a .70 cutoff for any of the pair-wise comparisons with a significant Θ_I _and Θ_ii _value are shown. All amino acid changes for Type II divergence are radical, indicating a change in amino acid properties; the single exception is indicated with 'C'.

## Discussion

### Distinct lineages of ferrichrome synthetases

Our phylogenetic analyses support the hypothesis that fungal ferrichrome synthetases fall into two distinct lineages corresponding to homologs of *C. heterostrophus *NPS2 and *A. nidulans *SidC. Some fungi contain representatives of both lineages while others lack a ferrichrome synthetase altogether. Significantly, ferrichrome NRPSs were not detected in any yeast species sampled (except the fission yeast, *S. pombe*), or in the zygomycetes *R. oryzae *and *P. blakesleeanus*, the ectomycorrhizal fungus *L. bicolor *or the chytrid *B. dendrobatitis*. While absence of a gene must be interpreted with caution, as genome sequences may be incomplete, the lack of the NPS1/SidC lineage in all Dothideomycetes (*C. heterostrophus, A. brassicicola, S. nodorum*, and *A. pullulans*) and Onygenales (*C. immitis, H. capsulatum*, and *U. reesii*), lack of the NPS2 lineage in Eurotiales (*Aspergillus *sp.), as well as a lack of any ferrichrome synthetase in all hemiascomycete yeasts, zygomycetes, or chytrids surveyed is likely significant.

The NPS1/SidC lineage predates the divergence of ascomycetes and basidiomycetes as its members are present in both of these groups. In contrast, the duplication into the two main NPS2 and NPS1/SidC lineages may have occurred in the ancestor of ascomycetes as the former lineage is only found within ascomycetes. The additional duplications within the NPS1/SidC lineage may have occurred also prior to the divergence of ascomycetes and basidiomycetes, as there are two distinct ferrichrome synthetase encoding genes from the NPS1/SidC lineage in both the basidiomycete *U. maydis *(Umfer3 and Umsid2) and the ascomycete *B. cinerea *(BC1G10928 and BC1G15494). This scenario would postulate an unlikely loss of one or the other of these genes in the majority of species examined. The other possibility is independent duplication of the NPS1/SidC type gene in certain species e.g., *U. maydis *and *B. cinerea*. However, in both ML and Bayesian phylogenetic analyses, the ascomycete proteins *B. cinerea *BC1G15494 and *F. graminearum *FG11026 grouped with, or outside of, basidiomycete proteins, suggesting an ancestral duplication of this lineage (Fig. 3, see Additional files 3 and 6).

It is possible that the duplications within the NPS1/SidC lineage may be associated with production of different ferrichromes. *F. graminearum *NPS1 (FG11026), has recently been shown to produce malonichrome (two GLY, one ALA) (G Adam, BG Turgeon, unpublished) while certain other ascomycete members (e.g., *A. nidulans *SidC) of the NPS1/SidC lineage produce ferricrocin (two GLY, one SER). The two ferrichrome synthetases in *U. maydis *also produce distinct products; Umfer3 produces ferrichrome A (two SER, one GLY) and Umsid2 produces ferrichrome (3 GLY).

### Evolution of domain architecture

**Figure 8:**
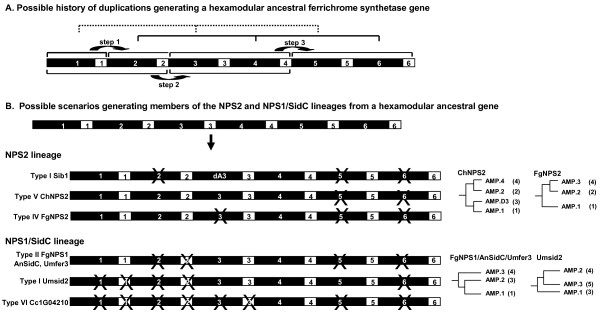
**Models for evolution of a hexamodular ancestral ferrichrome synthetase gene and for generation of domain architectures of the extant types examined in this study**. A. Possible origin of a hexamodular ancestral ferrichrome synthetase gene. We propose that a hexamodular gene arose by a series of duplication events. Step one: module 1 duplicates, forming module 1 and new module 2. Step two: modules 1 and 2 duplicate together, forming modules 1 and 2, and new modules 3 and 4. Step three: modules 3 and 4 duplicate together, forming modules 3 and 4, and new modules 5 and 6. This scenario predicts that modules 1, 3, and 5 (dotted lines) will show greater similarity to each other than to other modules. Similarly, modules 2, 4, and 6 (solid lines) will show greater similarity to each other than to modules 1, 3 and 5. B. Possible scenarios generating members of the NPS2 and NPS1/SidC lineages from a hexamodular ancestral gene. Trees to the right show relationships of extant AMP domains, based on Fig. 3. Numbers in parentheses indicate corresponding domain of hypothetical ancestral gene. Left side of figure indicates proposed losses of A (black boxes) or C (white boxes) domains, resulting in the extant gene.

In some respects, the C domain alone or in combination with the T domain can be considered the minimal evolutionary unit for NRPSs, as T-C units clearly occur in the absence of A domains. T-C units may also be considered the minimal functional units for NRPS synthesis as they can be charged by nonadjacent A domains [4,49,6,17,48]. T-C units lacking an associated A domain could be created either through independent duplication of T-C units or through loss of an associated A domain from a complete A-T-C module. If complete A-T-C module repeats arise by tandem duplication, the C domain phylogenies may provide a more complete picture of the evolutionary history of duplications at the locus. The relationships observed between C domains of modules 3 and 5 and among modules 2, 4, and 6 of the NPS2 lineage (Fig. 5A) and the partially ordered duplication history predicted by *C. heterostrophus *NPS2 C domains (Fig. 6Aii) imply a series of tandem duplication events involving single or double complete A-T-C units as a possible hypothesis for the evolution of a hexamodular ferrichrome synthetase NRPS (Fig. 6Aiv, Fig. 8). These events would occur as follows: Step 1) duplication of module 1 to form a bimodular gene, Step 2) duplication of the bimodular gene (modules 1 and 2) to form a tetramodular gene (modules 1–4), and Step 3) duplication of modules 3 and 4 to form a hexamodular gene (modules 1–6) (Fig. 8A, 6A).

These interpretations are based on algorithms for which it is assumed that there is no loss and no recombination, criteria that are clearly violated here for ferrichrome synthetases. We propose, however, that the C domains of *C. heterostrophus *NPS2 likely represent the full evolutionary history of ferrichrome synthetase modules. The chemical structure of ferrichromes (3 AA and 3 AHO) provides support for the notion of an ancestral gene with six complete modular units. Furthermore, our analyses (unpublished) and others [4] show little evidence for recombination within C domains. The tandem duplication hypothesis is based on these assumptions and is presented as one possible explanation for the diverse domain architectures. The phylogenetic relationships observed among A and C domains in both lineages are consistent with this proposed tandem duplication history if one postulates the loss of module 5 and 6 A domains from both lineages and the additional loss of the complete module 2 (A-T-C) from the SidC lineage (Figs. 6Aiii–v and 6Biii–v with losses shown in red, Figs. 4A, B). If these duplications occurred before the divergence of the majority of species examined, as supported by the reconciliation analysis, this scenario predicts that domains of modules 1, 3, and 5 (Fig. 8A, top, dotted lines, Figs. 6Av and 6Bv) will show greater similarity to each other than to other modules, as will modules 2, 4, and 6 (Fig. 8A, top, solid lines, Figs. 6Av and 6Bv).

In general, these predictions are supported when the relationships of A or C domains from each lineage are examined. In particular, the relationships between modules 3 and 5 and between 4 and 6, which would have resulted from the final duplication are more strongly supported (Figs. 4A, B, see Additional files 4A–D and 6). The results are not consistent with recent independent duplication of T-C units giving rise to the final T-C repeat in most ferrichrome synthetases (Fig. 2) as this latter mechanism would predict a closer relationship among C domains of modules 4, 5, and 6 which is not supported by C trees from either lineage. Instead, our analyses support the hypothesis of a hexamodular ancestor with six complete A-T-C modules, proposed previously by Schwecke [6], followed by loss of either complete A-T-C modules or individual A domains as the best hypothesis for the generation of the diverse domain architectures of the six ferrichrome synthetase domain structural types (Fig. 8). In the NPS2 lineage, for example, both *C. heterostrophus *(representative of Type V) and *F. graminearum *(representative of Type IV) have 6 C domains, although they have only 4 and 3 A domains, respectively. Analyses of C domains of these proteins clearly indicate that the second C domain of Types V and IV are related (Fig. 4, see Additional files 4A–D and 6). The same is true for the third C domains. The difference in protein architecture in this region is presence/absence of an A domain between C2 and C3 (i.e., the *F. graminearum *gene appears to be missing the third A domain found in the *C. heterostrophus *protein). Similarly, the second C domain in sib1 from *S. pombe *groups with the second C domain in *C. heterostrophus *NPS2 but lacks the corresponding A domain (Fig. 4), suggesting loss of this domain in the *S. pombe *gene. Our data thus suggest that differential loss of A domains in different members of this lineage has resulted in the three distinct domain architectures. A recent study of the microcystin synthase gene cluster has shown recombination breakpoints within NRPS A domains suggestive of recurrent A domain replacement [4]. Our analyses suggest that homologous recombination could also lead to complete loss of A domains.

For the NPS1/SidC lineage, *F. graminearum *NPS1, *A. nidulans *SidC and *U. maydis *fer3 all have 5 C domains and 3 A domains. A and C domain analyses of this lineage clearly indicate that there is a one to one relationship for all A and all C domains (Fig. 4, see Additional files 4C–D and 6). Examination of Umsid2, however, indicates that it has 3 A domains, but only 4 C domains; the module 1 A domain is related to module 2 A domains of the other members of this group, while both module 2 and 3 A domains are related to the C-terminal modules of other proteins in this lineage. Similarly the C domains from Umsid2 modules 2, 3, 4 are related to the C domains of modules 3, 4, 5 of the rest of the NPS1/SidC lineage. Umsid2 lacks the complete N-terminal A-T-C module of other NPS1/SidC members and retains the A domain corresponding to the module 4 C domain that our scenario postulates has been lost in other members of this lineage.

These data thus support the hypothesis [6] that the extant genes may have evolved from a hexamodular (A-T-C) ancestor and that repeated and independent losses of A domains or complete A-T-C modules may have given rise to the diverse domain architecture types observed in extant species.

### Domain architecture and mechanism of biosynthesis

How do ferrichrome synthetases differing in domain architecture, biosynthesize nearly identical chemical products? Several authors have suggested that T-C repeats can be used iteratively [17,48,49]. For example, Schwecke et al [6] have proposed a mechanism by which the functions of the missing *S. pombe *sib1 A domain (which should accompany the second T-C) and the degenerate second A domain (Fig. 2) are assumed by the first A domain, which charges both the second and third C domains in *cis*, thus attaching the three glycines required for the ferrichrome product. Similarly, some of the NPS2 lineage Type IV synthetases are predicted to make ferricrocin which contains two glycines and one serine. We speculate that the first A domain of this protein is used iteratively to attach two glycines by charging the T-C repeat after the second complete module. *U. maydis *sid2 has only a single A domain predicted to code for glycine yet ferrichrome contains three glycines. Therefore, the first A domain must also be used iteratively. Similarly, the last A domain of Types II-V may also charge the final two T-C units at the C terminal ends of these proteins to assemble the three AHO groups that form the core iron binding group, common to all ferrichrome synthetases [6]. Interestingly, the *U. maydis *sid2 protein, which has only a single terminal T-C, contains two complete A-T-C modules predicted to charge AHO. This protein thus must utilize an alternate mechanism to produce the three required AHO units and perhaps represents an intermediate step between a hexamodular ancestral gene with three complete A-T-C modules coding for AHO and a completely iterative system with a single A-T-C module coding for AHO followed by a T-C repeat that is used iteratively. Thus, loss of A domains in these NRPSs is compensated, likely, by iterative charging of T-C units.

Type VI *C. cinerea *CC1G04120 is unusual in that it has only a single A domain and a T-C repeat. It is possible that this gene is incomplete due to assembly errors, or may function together with another NRPS to form the complete ferrichrome product. Alternatively, it may produce a product such as desdiserylglycerylferrirhodin (DDF) which consists of three AHO residues only.

The mechanisms controlling iterative use of NRPS domains are, to our knowledge, unknown. Here we observe that proteins with distinct domain architectures produce nearly identical chemical products. Iterative synthesis provides yet another flexible mechanism for NRPS biosynthesis.

### Substrate specificity

Structural modeling results suggest that general features of the binding pocket such as size, hydrophobicity, and charge may be more important in determining substrate recognition than residues at fixed positions within the cavity. In homology based modeling of substrate specificity, small errors in the alignment between the experimental and the model sequence can lead to significant errors in the modeled structure. For this reason, we used an alignment of several experimental structures to optimize our alignments. We found that the A domains included in this study were remarkably conserved structurally and we were able to identify several conserved residue-patterns and structural features which aligned well in all the structures and served as markers to anchor our alignment of the experimental sequences, particularly near the residues that are supposed to form the wall of the binding site (the code). With careful attention to the alignment, we found that residues associated with the 10 or 13 AA 'codes' predicted to be important in substrate choice vary considerably and do not show a consistent pattern for A domains predicted to code the same substrate (Table 2, see Additional file 5). Thus, we found that the string of amino acids at the proposed 'code' positions was unable to predict substrates for any fungal A domain examined in this study. The 10AA code was originally deduced by extracting residues at positions predicted to interact with the Phe substrate in the 1_AMU_A domain from a multiple sequence alignment and is based on the assumption that, because A domains of NRPSs and other adenylating enzymes show high structural similarity, the positions in the 1_AMU_A structure should be important for other substrates [9]. Recent studies, however, have shown that additional residues may be important for interacting with other substrates such as AHO [5,6].

Our results from structural modeling and evolutionary analyses of functional residues point to key fragments within the binding pocket which surround and connect the α-helix and β-strand structural elements of the pocket, as general regions important for specificity. Our analyses also identified residue positions in addition to the 10 AA code positions within these fragments (229, 230, 240, 243, 280, 320, 322, and 326) which line the substrate pocket and are either positioned such that their side chains may interact with a substrate or are involved in shaping the size of the binding pocket (Table 2). Our study confirms [9] that D and K residues at positions 235 and 517 respectively (Table 2), adjacent to the N-terminal amino and C-terminal carboxyl groups, are conserved across all the sequences examined, and that they serve the general function of holding the amino and carboxyl groups of an amino acid substrate in the binding pocket and are not involved in recognition of a specific amino acid substrate.

We speculate that the residue positions showing a significant signal for functional divergence which fall outside of the binding pocket region on the surface of the protein (246–257 and 305–314) could have a role in either protein-protein interactions or interactions between the two subunits of the NRPS protein. One subunit contains both the substrate and AMP binding pockets while the other subunit covers the opening to the binding sites (Fig. 7A). In the crystal structure of the related adenylating enzyme, acetyl CoA synthetase (1PG3_A), this second subunit may adopt two configurations in order to accomplish the two half-reactions of this enzyme: 1) adenylation of the substrate and 2) subsequent transfer to coenzyme A. Each configuration exposes a different set of residues to the active site [50,51]. A similar mechanism may operate in NRPSs. Residues 305–314 on the surface of the first subunit are not in a position to interact directly with the binding pocket, but could be involved in mediating interactions between the two subunits.

Thus, our results suggest that a rigid 'code' of specific amino acids at particular residue positions may not be the most reliable approach to predicting specificity of fungal NRPS A domains. Instead, the general chemical, physical, and structural features of the binding pocket may be more important. We conclude that methods of substrate prediction which evaluate chemical features of amino acids within these key regions may be better able to predict substrate specificity. Our findings await manipulation of key residues predicted to affect the chemical properties of the binding pocket, followed by examination of how this affects substrate choice.

## Conclusion

Our results demonstrate two distinct lineages of ferrichrome synthetases in fungi and suggest that these genes are restricted to fission yeast, filamentous ascomycetes, and basidiomycetes. Phylogenetic analyses of domain architectures supports the hypothesis that the distinct domain architectures observed derive from a hexamodular ancestral gene through loss of individual A domains or complete A-T-C modules and support a series of tandem duplication events of single or double A-T-C modules as the mechanism generating this hexamodular ancestor.

Analyses of substrate specificity show that the proposed 10AA code was unable to infer substrate specificity for these fungal A domains. Structural modeling and evolutionary analyses of functional residues suggest that additional positions may play a role in substrate specificity. Our results confirm that two positions of the code which are conserved across all sequences examined, D235 and K517, likely do not play a role in amino acid substrate choice but instead serve the important function of anchoring the substrate in the binding pocket through interaction with the amino and carboxyl groups respectively.

## Authors' contributions

KEB selected and performed most of the phylogenetic analyses. DRR performed the protein structural modeling. BGT directed the research. KEB and BGT wrote the manuscript, with input from DRR.

## Supplementary Material

Additional file 1 **Protein accession numbers used in this study**. GenBank accession numbers of proteins in this study cross referenced to genome IDs where available. Click here for file

Additional file 2 **Species tree**. Species tree used for reconciliation analyses (See additional file 6). Click here for file

Additional file 3 **Bayesian analyses of all AMP domains examined in this study**. Alternative phylogenetic method to the maximum likelihood analysis of A domains from the complete dataset, provided for comparison (See additional file 6). Click here for file

Additional file 4 **Individual NPS2 and NPS1/SidC A and C domain lineage analyses**. The data provided represent separate phylogenetic analyses of NPS2 and NPS1/SidC adenylation and condensation domains (See additional file 6). Click here for file

Additional file 5 **Amino acids corresponding to the 10AA code positions of selected bacterial and fungal NRPS adenylation domains**. These data show the 10AA code for selected bacterial and fungal NRPS A domains described in the literature, or reported in this work, as coding for glycine, alanine, serine, or ornithine. Click here for file

Additional file 6 **Additional file**2, 3, 4**figure legends**. Figure legends for additional files 2, 3, 4. Click here for file
