# Supplementary material for: Module evolution and substrate specificity of fungal nonribosomal peptide synthetases involved in siderophore biosynthesis
Source: BMC Evol Biol. 2008 Dec 3;8:328. doi: 10.1186/1471-2148-8-328 (PMC2644324; doi:10.1186/1471-2148-8-328)
Supplement: Additional file 1 — Protein accession numbers used in this study. GenBank accession numbers of proteins in this study cross referenced to genome IDs where available. [file 1471-2148-8-328-S1.pdf]

**Additional File 1: Protein accession numbers used in this study**

| Species | GenBank Acc# | Genome ID [a] | Protein Name [b] | Reference |
|---|---|---|---|---|
| *Alternaria brassicicola* | ABU42595 | AB44259 | NPS2 | [1] |
| *Aspergillus fumigatus* | EAL92059 | Afu3g15270 | NRPS7 | [2] |
| | EAL86616 | Afu3g03350 | NRPS3 | [2] |
| | EAL91050 | Afu3g17200 | NRPS2 (SidC) | [2][3] |
| *Aspergillus nidulans* | XP_753088 | AN0607.3 | SidC | [3] |
| *Aspergillus niger* | XP_001390952.1 | Aspni1_207636 | hypothetical protein | |
| *Aspergillus oryzae* | BAE59066 | AO9002300528 | Sid2/NRPS36 | [2] |
| *Aspergillus terreus* | XP_001212122.1 | ATEG02944.1 | NRPS83 | [2] |
| | XP_001217069.1 | ATEG08448.1 | NRPS82 | [2] |
| | XP_001214251.1 | ATEG05073 .1 | NRPS71/ SidC | [2] |
| *Aureobasidium pullulans* | AAD00581 | | peptide synthetase | [4] |
| *Botrytis cinereus* | XP_001550755.1 | BC1G10928.1 | hypothetical protein | |
| | XP_001557929.1 | BC1G03511.1 | hypothetical protein | |
| | XP_001546022.1 | BC1G15494.1 [d] | hypothetical protein | |
| *Chaetomium globosum* | XP_001228767 | CHGG02251.1 | hypothetical protein | |
| | XP_001226019.1 | CHGG10752.1 | hypothetical protein | |
| | XP_001230007.1 | CHGG03491.1 | hypothetical protein | |
| | XP_001227470.1 | CHGG09543.1 | hypothetical protein | |
| *Coccidioides immitis (RS)* | XP_001247170.1 | CIMG00941.1 | hypothetical protein | |
| *Cochliobolus heterostrophus* | AAX09983 | | NPS1 | [5] |
| | AAX09984 | | NPS2 | [5] |
| | AAX09985 | | NPS3 | [5] |
| | AAX09986 | | NPS4 | [5] |
| | AAX09987 | | NPS5 | [5] |
| | AAX09988 | | NPS6 | [5] |
| | AAX09989 | | NPS7 | [5] |
| | AAX09990 | | NPS8 | [5] |
| | AAX09994 | | NPS9 | [5] |
| | AAX09992 | | NPS10 | [5] |
| | AAX09993 | | NPS11 | [5] |
| | AAX09994 | | NPS12 | [5] |
| | AY884198 | | NPS13 | [5] |
| *Coprinus cinerea* | EAU88504.1 | CC1G04210.1 | hypothetical protein | |
| *Fusarium graminearum* | XP_391202.1 | FG11026.2 | NPS1 | [1] |
| | XP_385548.1 | FG05372.2 | NPS2 | [6] |
| *Histoplasma capsulatum* | XP_001544796.1 | HCAG01843.1 | hypothetical protein | |
| | XP_001538006.1<br>XP_001538007.1 | HCAG07428.1 [c, d]<br>HCAG07429.1 [c, d] | hypothetical protein | |
| *Magnaporthe grisea* | XP_001407762.1 | MGG12175.3 | SSM1 | [7] |
| *Neurospora crassa* | XP_960302 | NCU07119.2 | putative intracellular siderophore NPS | [8]<br>[4] |
| *Omphalotus olearius* | AAX49356 | | fso1 | [4] |
| *Schizosaccharomyces pombe* | CAB72227 | | sib1,SPAC23G3.02c | [4] |

| | | | | |
|---|---|---|---|---|
| *Sclerotinia sclerotiorum* | XP_001593263.1 | SS1G06185.1 | hypothetical protein | |
| | XP_001595604.1 | SS1G03693.1 | hypothetical protein | |
| *Stagonospora nodorum* | | SNU02134.1 | hypothetical protein | |
| *Trichoderma reesii* | | 69946 (JGI) | hypothetical protein | |
| *Uncinocarpus reesii* | | UREG00890.1 [c]<br>UREG00891.1 [c] | hypothetical protein | |
| *Ustilago maydis* | XP_757581.1 | UM01434.1 | fer3 | [9] |
| | AAB93493 | UM05165.1 | sid2 | [10] |
| *Erwinia carotovora subsp. atroseptica* | YP_049592 | | nonribosomal peptide synthetase | |

[a] Source as indicated in Materials and Methods
[b] Common name
[c] Two genes reannotated as a single gene
[d] Incomplete gene

## References


1. Oide S, Krasnoff SB, Gibson DM, Turgeon BG: **Intracellular siderophores are essential for ascomycete sexual development in heterothallic *Cochliobolus heterostrophus* and homothallic *Gibberella zeae***. *Eukaryotic Cell* 2007, **6**(8):1339-1353.
2. Cramer RA, Stajich JE, Yamanaka,Y, Dietrich FS, Steinbach, W, Perfect, JR: **Phylogenomic analysis of non-ribosomal peptide synthetases in the genus *Aspergillus***. *Gene* 2006, **383**:24-32.
3. Eisendle M, Oberegger H., Zadra I, and Haas H: **The siderophore system is essential for viability of *Aspergillus nidulans:* functional analysis of two genes encoding l-ornithine N 5-monooxygenase (sidA) and a non-ribosomal peptide synthetase (sidC)**. *Molecular Microbiology* 2003, **49**:359-375.
4. Schwecke T, Goettling K, Durek P, Duenas I, Kaeufer NF, Zock ES, Staub E, Neuhof T, Dieckmann R, von Doehren H: **Nonribosomal peptide synthesis in *Schizosaccharomyces pombe* and the architectures of ferrichrome-type siderophore synthetases in fungi**. *Chembiochem* 2006, 7:612-622.
5. Lee B, Kroken, S, Chou DYT, Robbertse B, Yoder OC, and Turgeon BG: **Functional analysis of all nonribosomal peptide synthetases in *Cochliobolus heterostrophus* reveals a factor, NPS6, involved in virulence and resistance to oxidative stress** *Eukaryotic Cell* 2005, **4**(3):545-555.
6. Tobiasen C, Aahman J, Ravnholt KS, Bjerrum, MJ, Grell MN, and Giese H: **Nonribosomal peptide synthetase (NPS) genes in *Fusarium graminearum, F. culmorum* and *F. pseudograminearium* and identification of NPS2 as the producer of ferricrocin**. *Current Genetics* 2007, **51**:43-58.
7. Hof C, Eisfeld K, Welzel K, Antelo L, Foster AJ, Anke H: **Ferricrocin synthesis in *Magnaporthe grisea* and its role in pathogenicity in rice**. *Molecular Plant Pathology* 2007, **8**(2):163-172.
8. Galagan JE, Calvo SE, Borkovich KA, Selker EU, Read ND, Jaffe D, FitzHugh W, Ma LJ, Smirnov S, Purcell S *et al*: **The genome sequence of the filamentous fungus *Neurospora crassa***. *Nature* 2003, **422**(6934):859-868.

9. Eichhorn H, Lessing F, Winterberg B, Schirawski J, Kamper J, Muller P, Kahmann R: **A ferroxidation/permeation iron uptake system is required for virulence in *Ustilago maydis***. *Plant Cell* 2006, **18**(11):3332-3345.

10. Yuan WM, Gentil GD, Budde, AD, and Leong SA: **Characterization of the *Ustilago maydis sid2* gene, encoding a multidomain peptide synthetase in the ferrichrome biosynthetic gene cluster**. *Journal of Bacteriology* 2001, **183**(13):4040-4051.
