# Supplementary figures and images for: Module evolution and substrate specificity of fungal nonribosomal peptide synthetases involved in siderophore biosynthesis
Source: BMC Evol Biol. 2008 Dec 3;8:328. doi: 10.1186/1471-2148-8-328 (PMC2644324; doi:10.1186/1471-2148-8-328)

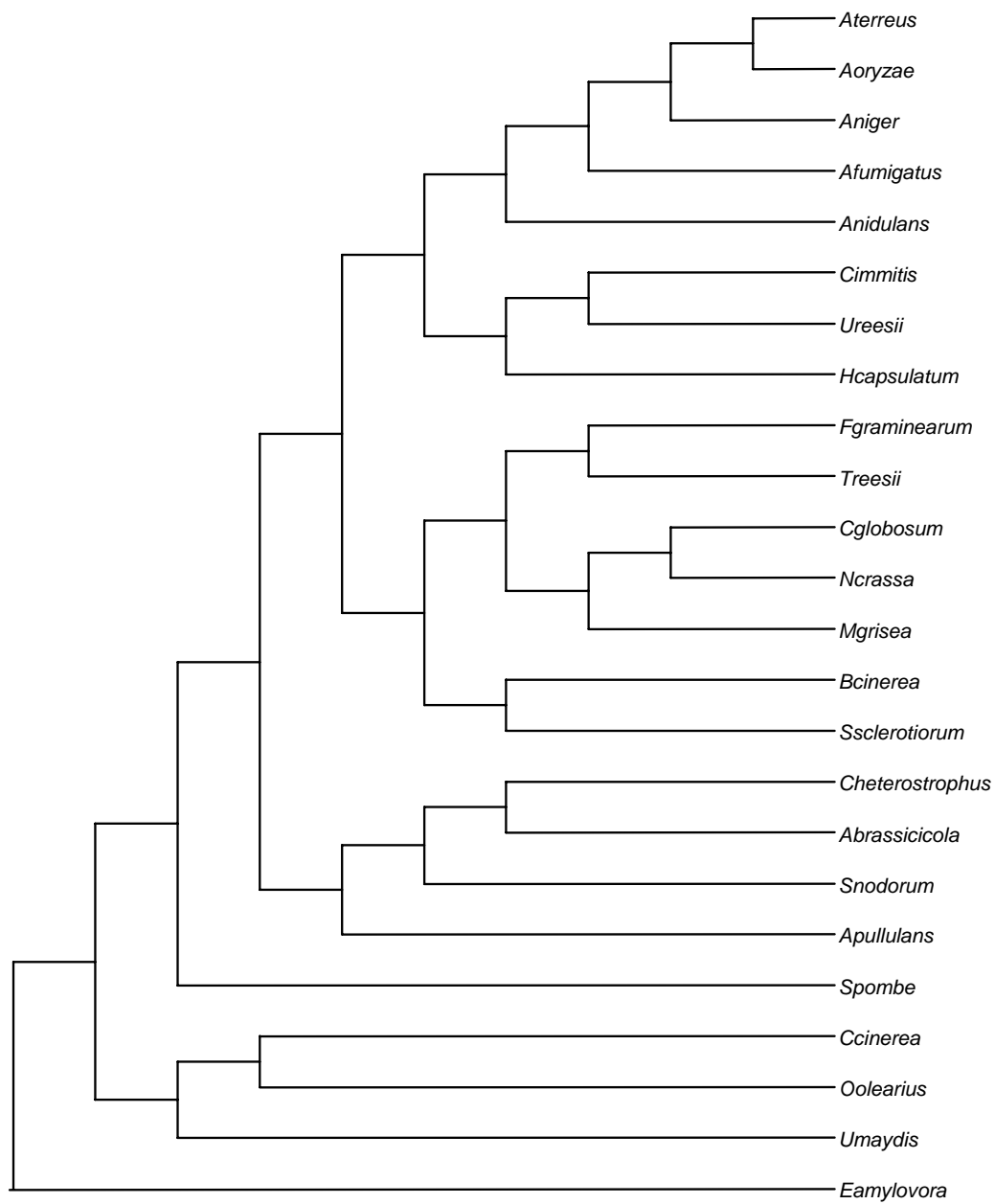

**Additional File 2. Species tree.**

Supplement: Additional file 2 — Species tree. Species tree used for reconciliation analyses (See additional file 6). [file 1471-2148-8-328-S2.pdf]

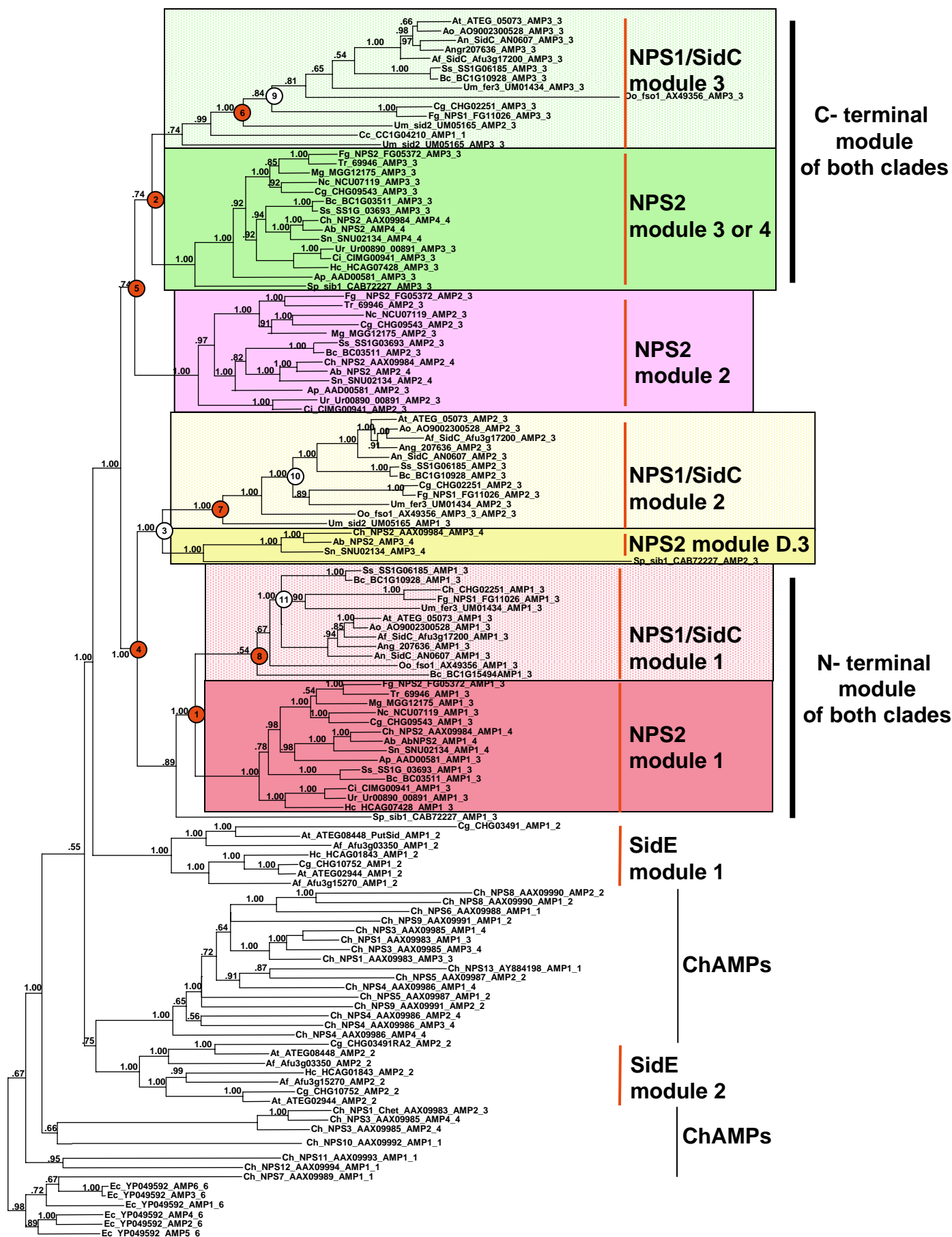

Supplement: Additional file 3 — Bayesian analyses of all AMP domains examined in this study. Alternative phylogenetic method to the maximum likelihood analysis of A domains from the complete dataset, provided for comparison (See additional file 6). [file 1471-2148-8-328-S3.pdf]
