# Supplementary material for: Module evolution and substrate specificity of fungal nonribosomal peptide synthetases involved in siderophore biosynthesis
Source: BMC Evol Biol. 2008 Dec 3;8:328. doi: 10.1186/1471-2148-8-328 (PMC2644324; doi:10.1186/1471-2148-8-328)
Supplement: Additional file 4 — Individual NPS2 and NPS1/SidC A and C domain lineage analyses. The data provided represent separate phylogenetic analyses of NPS2 and NPS1/SidC adenylation and condensation domains (See additional file 6). [file 1471-2148-8-328-S4.pdf]

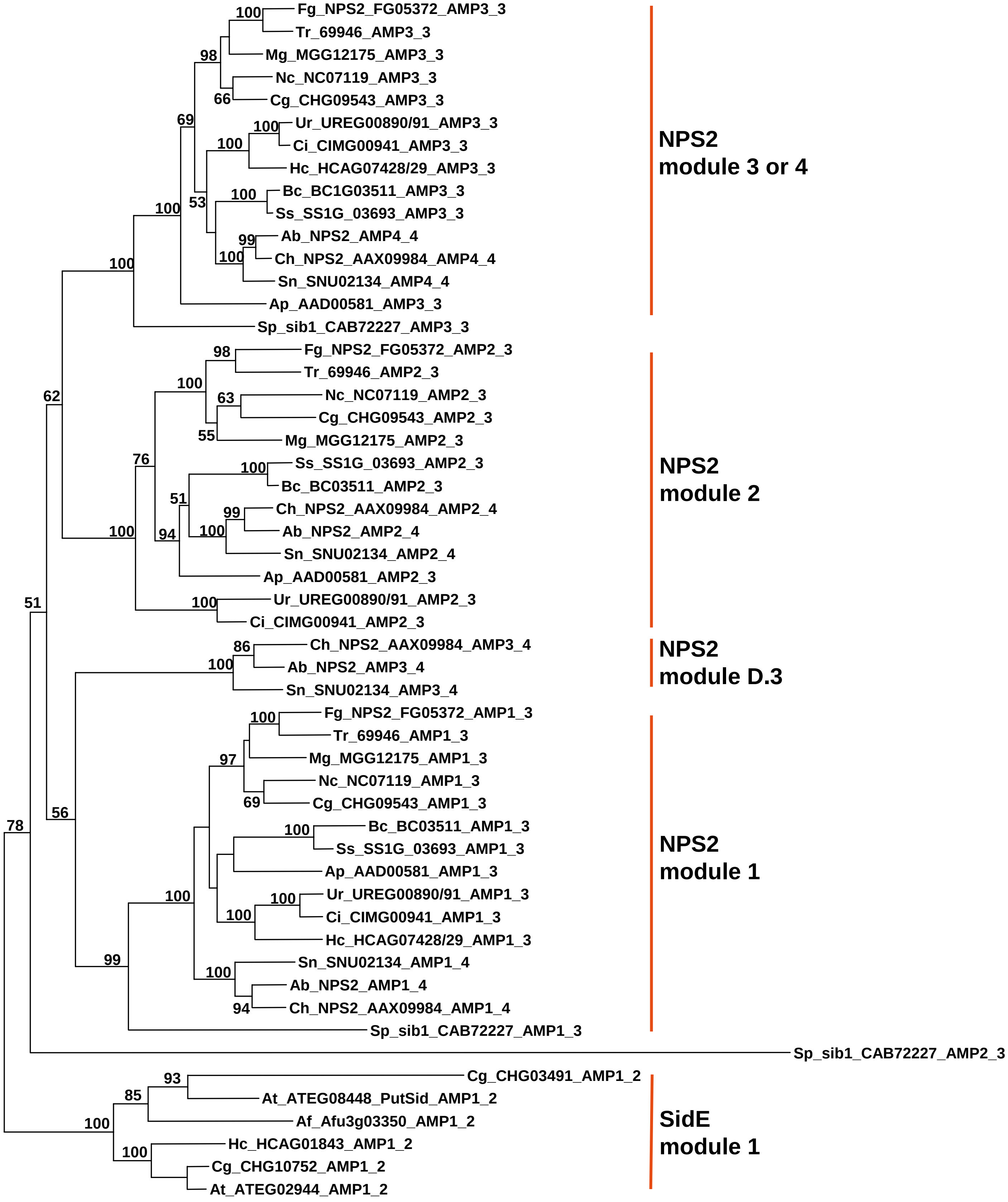

Additional File 4Ai. NPS2 lineage AMP domains. Maximum Likelihood

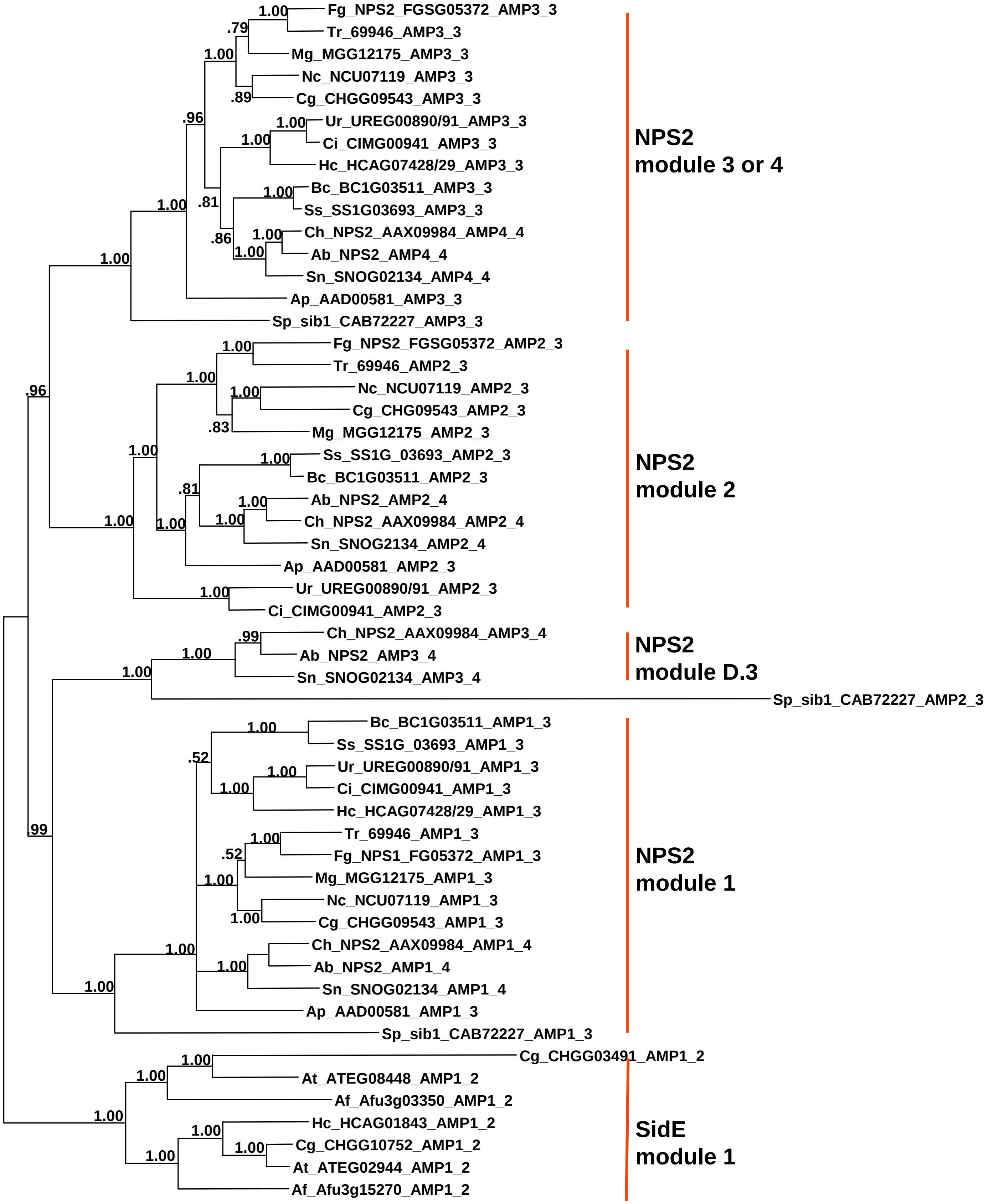

Additional File 4Aii. NPS2 lineage AMP domains. Bayesian

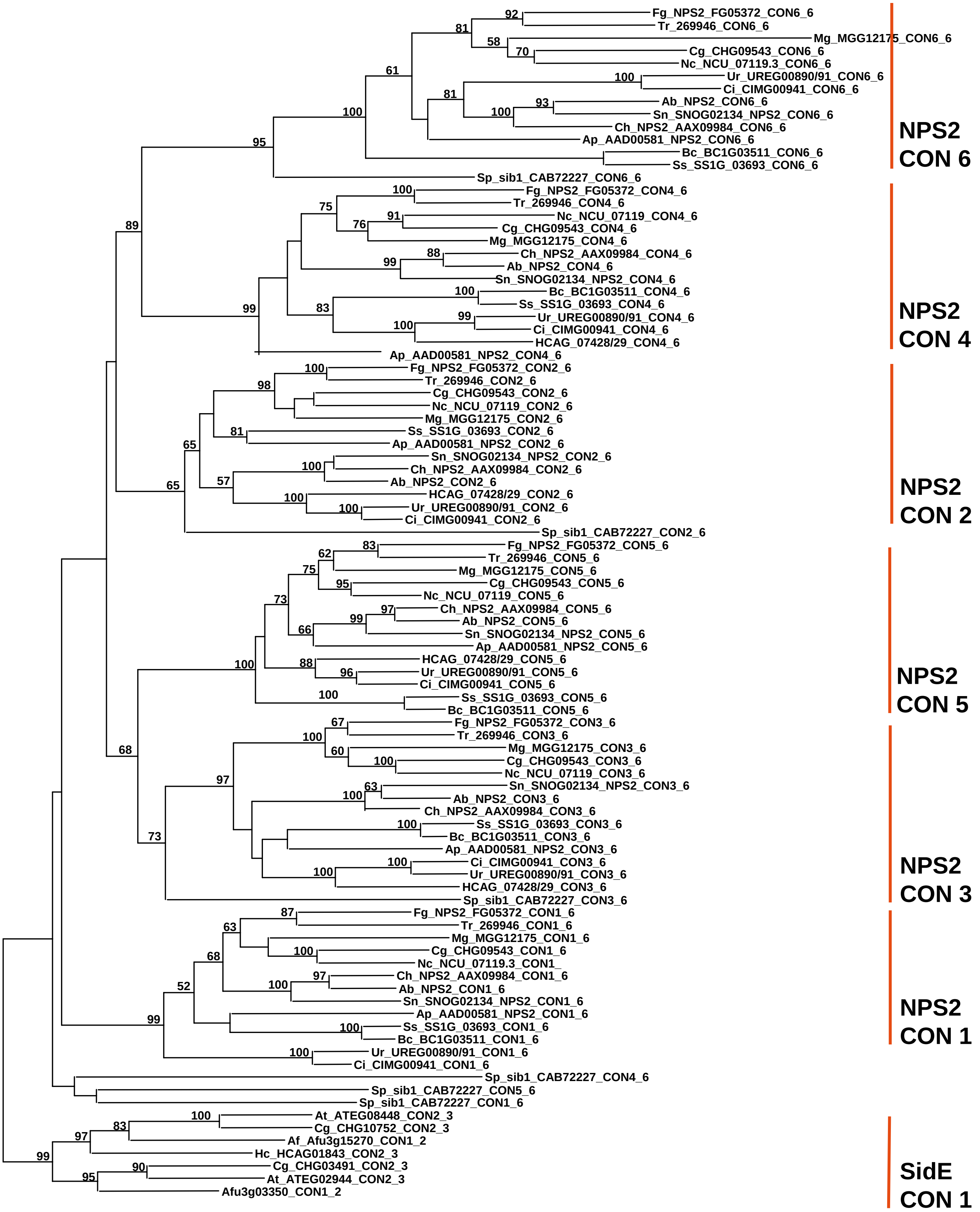

Additional File 4Bi. NPS2 lineage CON domains. Maximum Likelihood

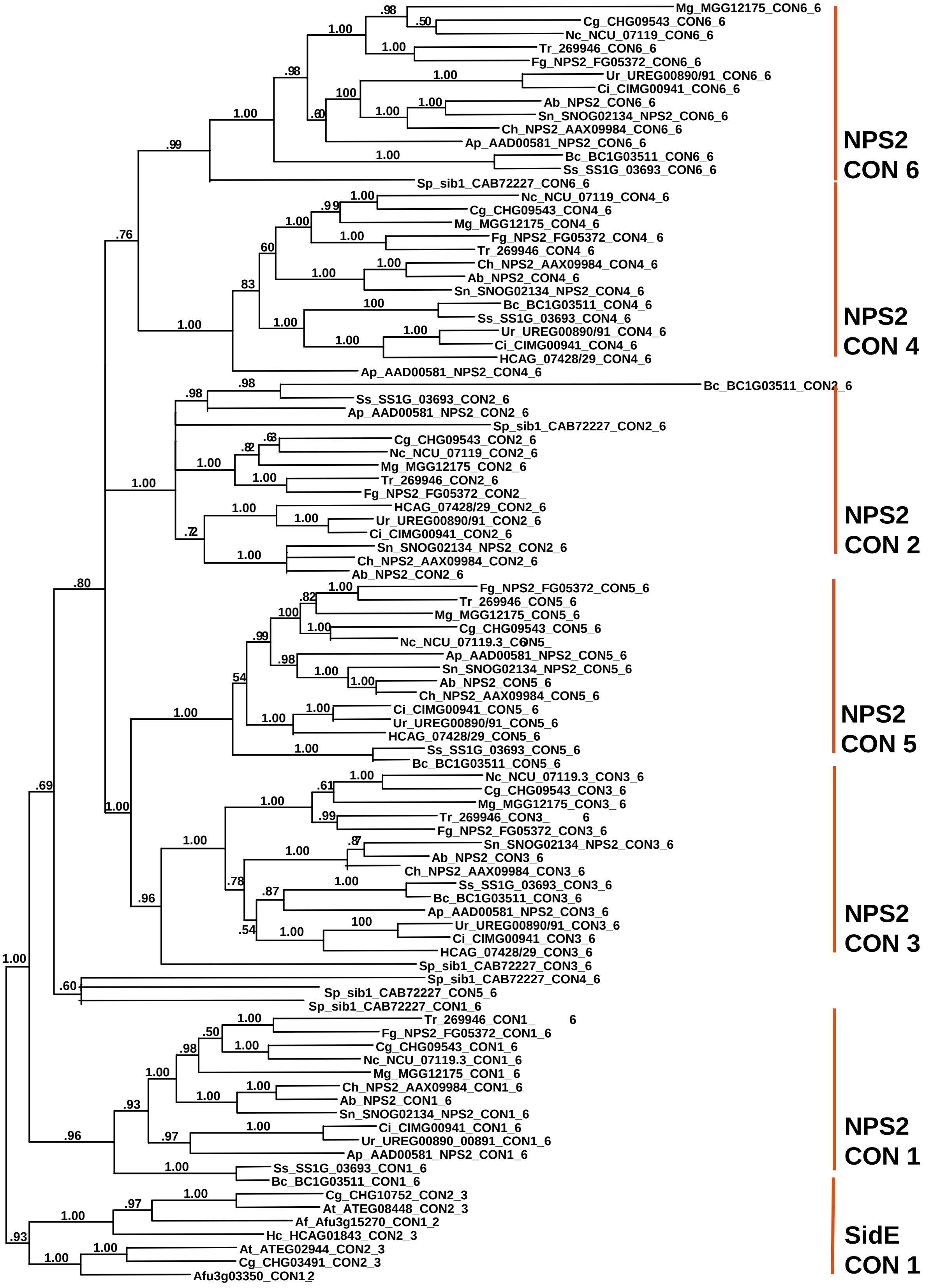

Additional File 4Bii. NPS2 lineage CON domains. Bayesian

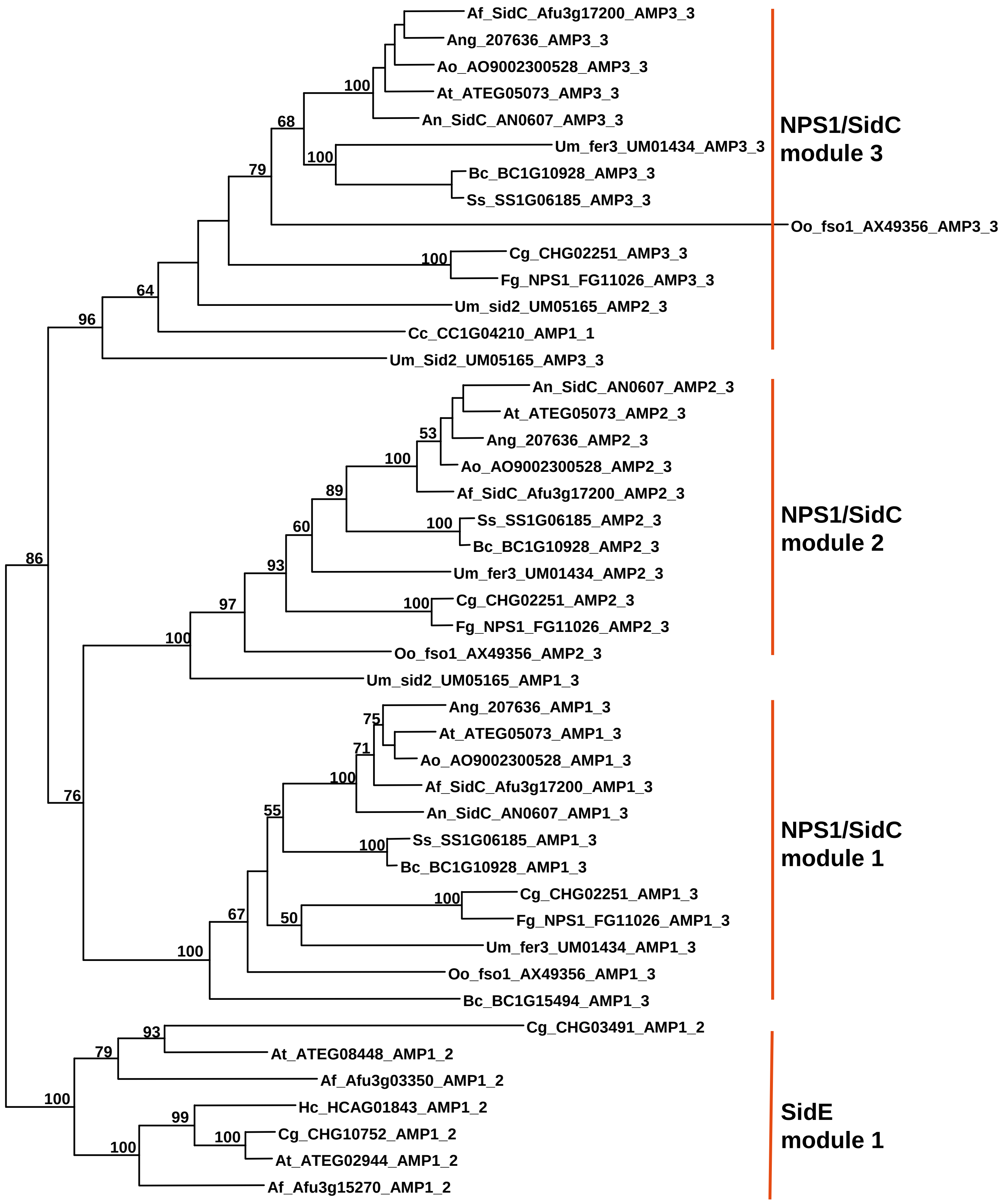

Additional File 4Ci. NPS1/SidC lineage AMP domains. Maximum Likelihood

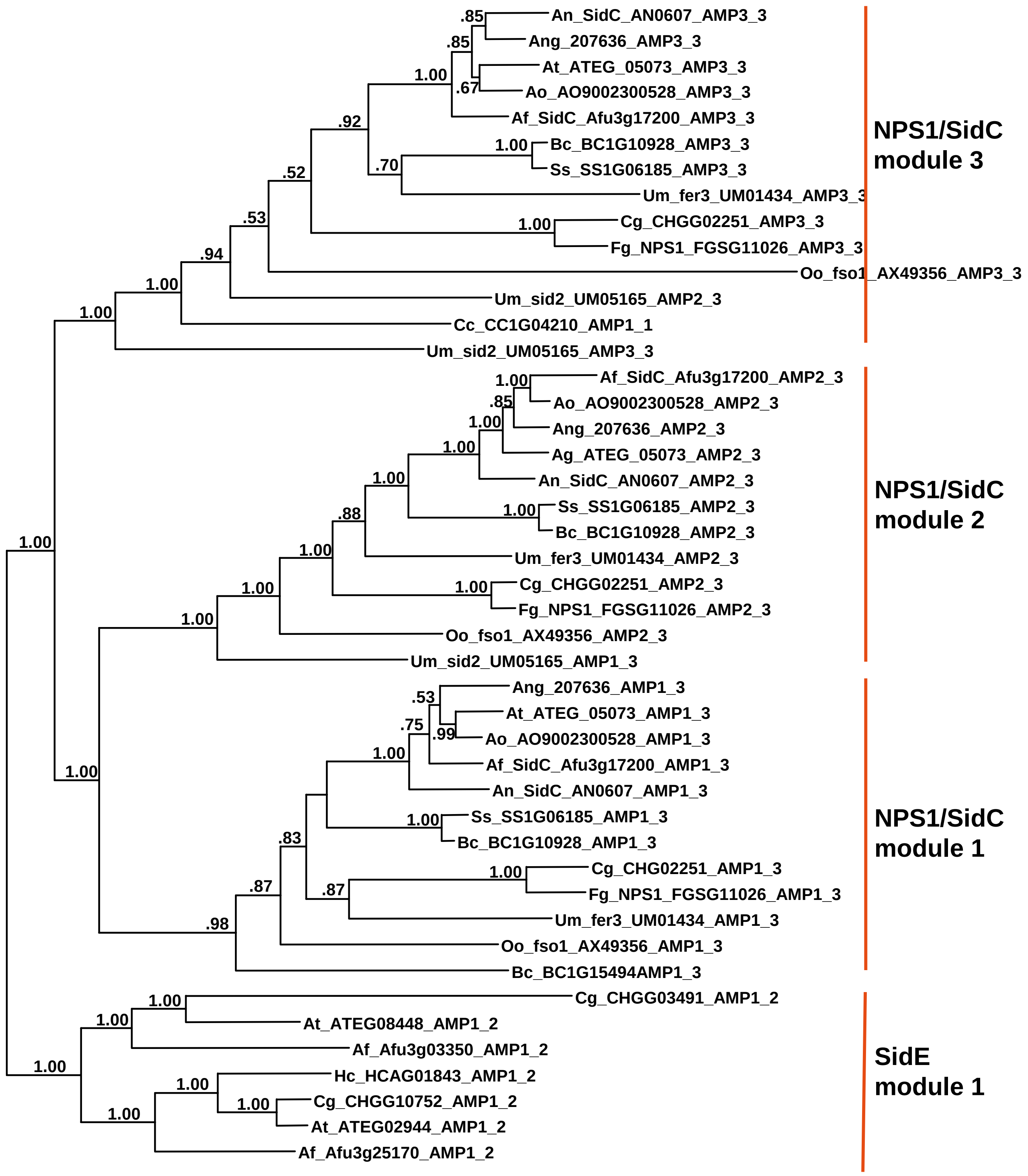

Additional File 4Cii. NPS1/SidC lineage AMP domains. Bayesian

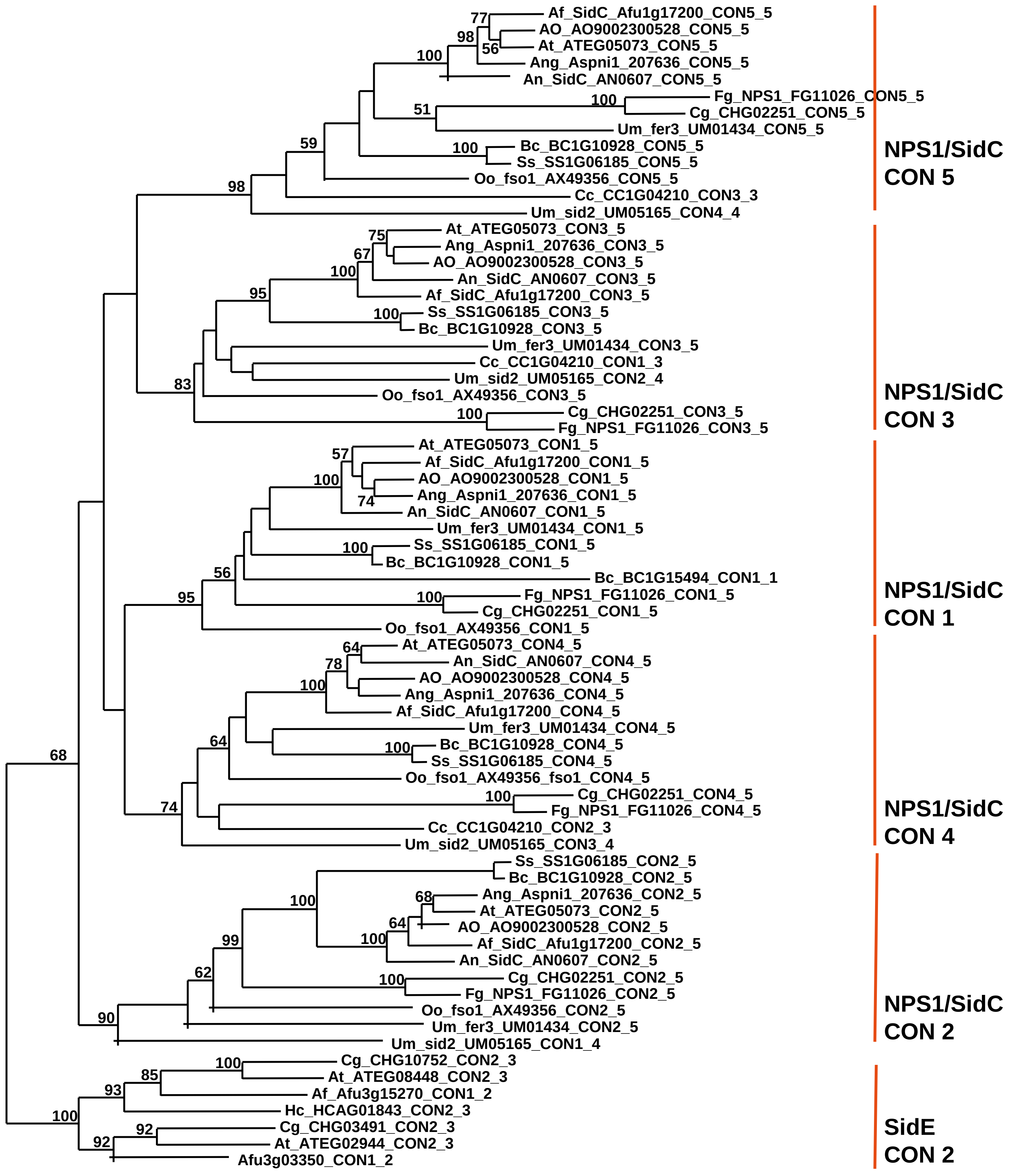

Additional File 4Di. NPS1/SidC lineage CON domains. Maximum Likelihood

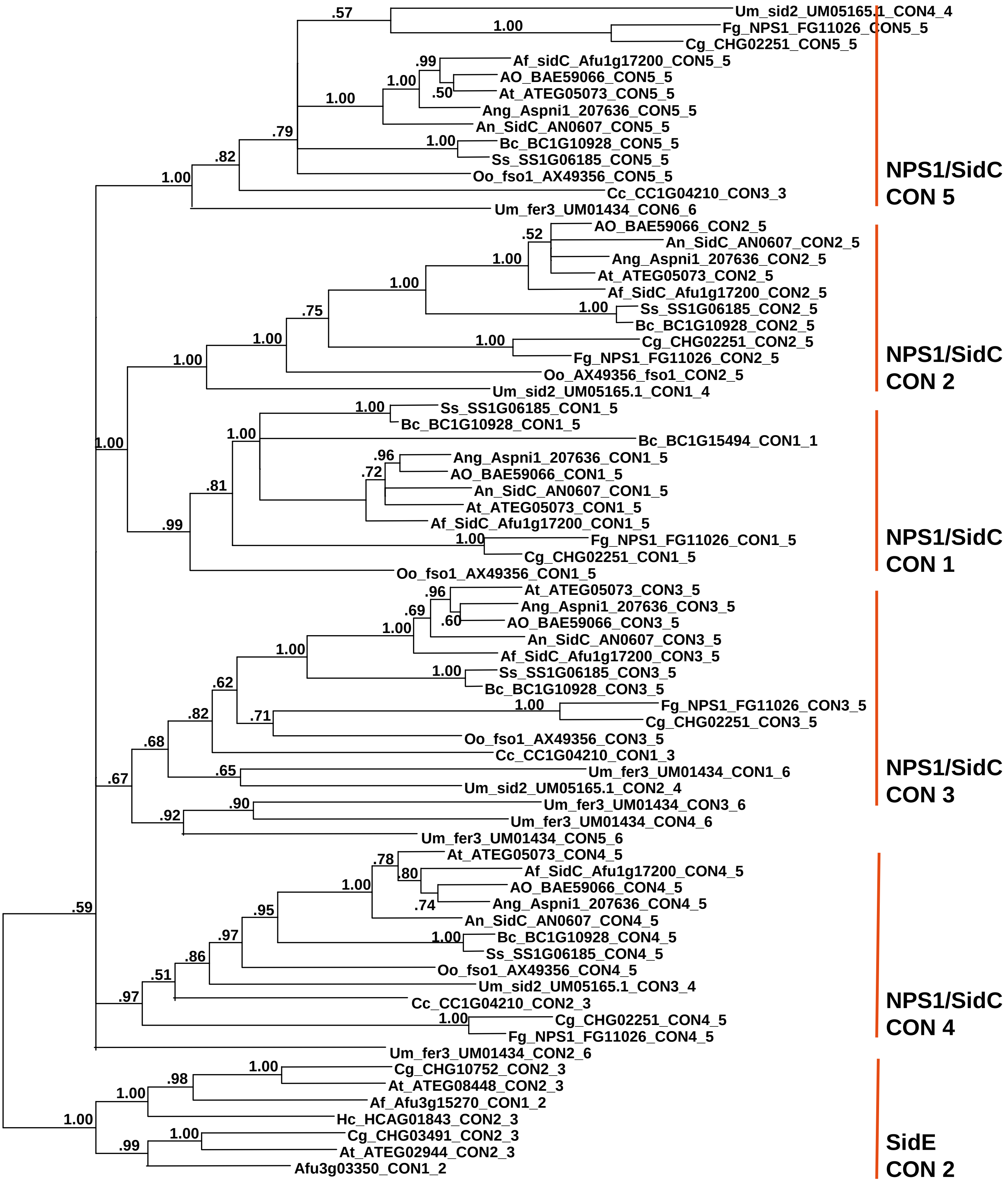

Additional File 4Dii. NPS1/SidC lineage CON domains. Bayesian
