# Supplementary material for: Module evolution and substrate specificity of fungal nonribosomal peptide synthetases involved in siderophore biosynthesis
Source: BMC Evol Biol. 2008 Dec 3;8:328. doi: 10.1186/1471-2148-8-328 (PMC2644324; doi:10.1186/1471-2148-8-328)
Supplement: Additional file 5 — Amino acids corresponding to the 10AA code positions of selected bacterial and fungal NRPS adenylation domains. These data show the 10AA code for selected bacterial and fungal NRPS A domains described in the literature, or reported in this work, as coding for glycine, alanine, serine, or ornithine. [file 1471-2148-8-328-S5.pdf]

Additional File 5. Amino acids corresponding to the 10AA code positions of selected bacterial and fungal NRPS adenylation domains.

AminoAcid Substrate	Species	Accession #	NRPS/AMP domain[a]	10AA code position										Reference
				235	236	239	278	299	301	322	330	331	517	
**GLYCINE**														
fungal:	*Hypocrea virens*	Q8NJX1	TEX1_AMP2_18	D	I	G	M	V	V	G	V	L	**K**	[1, 2]
	Tolypocladium inflatum	Q09164	TOLIN_AMP7_11	D	I	Q	M	F	V	A	M	Q	K	[3, 4]
	Schizosaccharomyces pombe	SPAC23G3.02c*	Sib1_AMP1_3	D	V	F	D	I	I	A	I	H	K	This study
	Ustilago maydis	UM05165.1**	sid2_AMP1_3	D	L	M	L	I	G	L	L	I	K	This study
	Cochliobolus heterostrophus	AAX09984*	NPS2_AMP4_4	D	M	Y	D	Y	I	S	F	C	K	This study
bacterial:	*Bacillus subtilis*	P45745	DHBF_AMP1_2	D	I	S	Q	L	G	L	I	W	K	[5]
	Myxococcus xanthus	Q50858	SafAMx1_AMP1_2	D	I	L	Q	L	G	L	V	W	K	[5]
	Bacillus cereus	Q81DQ0	GlycineAMPLigase_AMP1_1	D	I	L	Q	L	G	L	I	W	K	[5]
	Bacillus anthracis	Q81QP7	DHBF_AMP1_2	D	I	L	Q	L	G	L	I	W	K	[5]
	Streptomyces chrysomallus	Q9L8H4	ActinoIII_AMP2_3	D	I	L	Q	L	G	L	I	W	K	[5]
	Nostoc sp.	Q9RAH2	NosC_AMP2_3	D	I	L	Q	L	G	L	I	W	K	[5]
			Stachelhaus [2] consensus	none										
**ALANINE**														
fungal:	*Claviceps purpurea*	O94205	LPS1_AMP1_3	D	L	F	F	C	G	G	P	L	K	[2, 6-8]
	Hypocrea virens	Q8NJX1	Tex1_AMP3_18	D	V	G	F	V	A	G	V	L	K	[9]
	Hypocrea virens	Q8NJX1	Tex1_AMP8_18	D	I	F	V	V	A	G	V	I	K	[9]
	Cochliobolus carbonum	Q01886	HTS1_AMP2_4	D	A	G	G	C	A	M	V	A	K	[2, 10]
	Tolypocladium inflatum	Q09164	SimA_AMP11_11 (CssA)	D	V	F	I	Y	A	A	I	L	K	[2-4].
	Cochliobolus carbonum	Q01886	HTS1_Ccarb_AMP3_4	D	L	L	F	F	I	S	V	L	K	[2, 10]
	Tolypocladium inflatum	Q09164	SimA_AMP1_11 (CssA)	D	L	W	F	Y	I	A	V	V	K	[2-4]
bacterial:	*Streptococcus agalactiae*	P59591	DLTA_AMP1_1	D	L	M	T	F	D	A	V	A	K	[5]
	Myxococcus xanthus	Q50857	SafBMx1_AMP1_1	D	L	F	N	L	A	L	T	Y	K	[5][2]
	Streptococcus pneumoniae	P0A398	DLTA_AMP1_1	D	L	M	T	F	D	A	V	A	K	[5]
	Lactobacillus rhamnosus	P35854	DLTA_AMP1_1	D	L	M	V	F	C	T	V	A	K	[5]
	Bacillus subtilis	P39581	DLTA_AMP1_1	D	L	M	T	F	C	T	V	A	K	[5]
	Staphylococcus aureus	P68876	DLTA_AMP1_1	D	L	M	V	F	C	T	V	A	K	[5]
			Stachelhaus [2] consensus	D	L	L	F	G	I	A	V	L	K	[2]
**ORNITHINE**														
fungal:	*Claviceps purpurea*	O94205	LPS1_1_AMP2_3	D	L	V	G	M	A	A	V	G	K	[2, 8, 11]
	Schizosaccharomyces pombe	SPAC23G3.02c*	Sib1_AMP3_3	D	V	L	D	I	G	F	I	G	K	This study
(AHO)	*Cochliobolus heterostrophus*	AAX09984*	NPS2_AMP4_4	D	V	L	D	I	G	G	I	G	K	This study
(AHO)	*Fusarium graminearum*	FG05372.1**	NPS2_AMP3_3	D	V	L	D	I	G	A	I	G	K	This study
(AHO)	*Aspergillus nidulans*	AN0607.3**	SidC_AMP3_3	D	P	L	S	T	G	A	I	G	K	This study
(AHO)	*Fusarium graminearum*	FG11026.1**	NPS1_AMP3_3	D	P	T	G	T	G	F	I	G	K	This study
(AHO)	*Ustilago maydis*	UM05165.1**	sid2_AMP3_3	D	V	I	D	M	G	A	I	G	K	This study

bacterial:	*Brevibacillus parabrevis*	O30409	TycC_3_AMP5_6	D	V	G	E	I	G	S	I	D	K	[5]
	Bacillus licheniformis	O68007	BACB_AMP2_2	D	V	G	E	I	G	S	V	D	K	[5]
	Mycobacterium smegmatis	O87313	FxbB_AMP1_2	D	I	N	Y	W	G	G	I	G	K	[5]
	Mycobacterium smegmatis	O87314	FxbC_AMP1_4	D	M	E	N	L	G	L	I	N	K	[5][2]
	Mycobacterium smegmatis	O87314	FxbC_AMP3_4	D	M	E	N	L	G	L	I	N	K	[2, 5]
	Bacillus subtilis	O87606	FenC_AMP2_2	D	V	G	E	I	G	S	I	G	K	[2, 5]
	Aneurinibacillus migulanus	P0C063	GRSB_AMP3_4	D	V	G	E	I	G	S	I	D	K	[2, 5]
	Bacillus subtilis	P39845	PPS1_AMP2_2	D	V	G	E	I	G	S	I	D	K	[5]
			Stachelhaus [2] consensus Orn (1)	D	M	E	N	L	G	L	I	N	K	[2]
			Stachelhaus [2] consensus Orn (2)	D	V	G	E	I	G	S	I	D	K	[2]
**SERINE**														
fungal:	*Hypocrea virens*	Q8NJX1	Tex1_AMP_AMP10_18	D	V	G	Y	L	A	A	V	Y	K	[2]
bacterial:	*E. coli*	P11454	EntF_AMP1_1	D	V	V	H	F	S	L	V	D	K	[2, 5]
	Bacillus subilis	Q9R9I9	MycC_AMP1_2	D	V	V	H	F	S	L	I	D	K	[5]
	Nostoc sp.	Q9RAH4	NosA_AMP2_4	D	V	V	H	I	S	L	I	D	K	[5]
	Microcystis aeruginosa	Q9RNB1	McyA_AMP1_2	D	V	V	H	F	S	L	I	D	K	[5]
	Streptomyces coelicolor	Q9Z4X6	CDA PS1__AMP1_6	D	V	V	H	F	S	L	V	D	K	[5]
	Pseudomonas syringae pv. syringae	AAC80285.1*	SYRE1_AMP1_2	D	L	V	H	L	S	L	I	D	K	[2]
	Pseudomonas syringae pv. syringae	AAC80285.1*	SYRE2_AMP2_2	D	V	V	H	L	S	L	I	D	K	[2]
			Stachelhaus [2] consensus	D	V	W	H	L	S	L	I	D	K	[2]

[a] NRPS A domains identified in the literature (referenced in final column) as coding for GLY, ALA, SER, or ORN were aligned with TCoffee using the GrsA 1AMU structure as template.   This alignment was inspected manually to insure consistency with a structural alignment of 1AMU and A domains from a number of other related A domains (1PG3_A, 1ULT_A, 1LC_I, 1T5D_X and 1MD9_A) and amino acids at positions corresponding to the 10AA code were extracted. The consensus “code” for each substrate determined by Stachelhaus et. al. [2] are shown.

Column 1. For ornithine, only a single representative is known from fungi. Domains identified in this study as activating AHO (N^5-acetyl-N^5-hydroxy-L-ornithine, N^5-acyl-N^5-hydroxy-L- ornithine), were included.

Column 3. All entries are uniprot (EMBL) accessions unless otherwise marked * = GenBank, ** = Broad Institute ID.

Column 4. NRPS name/A domain. For example, TEX1_AMP2_18 is the second A domain of a total of 18 in TEX1.

References

1. Wiest A, Grzegorski D, Xu BW, Goulard C, Rebuffat S, Ebbole DJ, Bodo B, Kenerley C: **Identification of peptaibols from *Trichoderma virens* and cloning of a peptaibol synthetase**. *J Biol Chem* 2002, **277**(23):20862-20868.
2. Stachelhaus T, Mootz, Henning D, and Marahiel M: **The specificity-conferring code of adenylation domains in nonribosomal peptide sythetases**. *Chem & Biol* 1999, **6**:493-505.
3. Husic DW, Husic HD, Tolbert NE: **The oxidative photosynthetic carbon cycle or C2 cycle**. *CRC Critical Rev Plant Sci* 1987, **5**:45-100.

4. Lawen A, Traber R: **Substrate specificities of cyclosporine synthetase and peptolide sdz 214-103 synthetase - comparison of the substrate specificities of the related multifunctional polypeptides**. *J Biol Chem*1993, **268**(27):20452-20465.
5. Rausch C, Weber, T, Kohlbacher, O, Wohlleben W, and Huson DH: **Specificity predictions of adenylation domains in nonribosomal peptide synthetases (NRPS) using transductive support vector machines (TSVMs)**. *Nucl Acids Res* 2005, **33**(18):5799-5808.
6. Tudzynski P, Holter K, Correia T, Arntz C, Grammel N, Keller U: **Evidence for an ergot alkaloid gene cluster in *Claviceps purpurea***. *Molecular and General Genetics* 1999, **261**(1):133-141.
7. Keller N, Tudzynski B: **Ergot Alkaloids**. In: *The Mycota, Industrial applications.* Edited by Osiewacz, HD. Berlin: Springer; 2001: 157-181.
8. Walzel B, Riederer B, Keller U: **Mechanism of alkaloid cyclopeptide synthesis in the ergot fungus *Claviceps purpurea***. *Chem & Biol* 1997, **4**(3):223-230.
9. Challis GL, Ravel J, Townsend CA: **Predictive, structure-based model of amino acid recognition by nonribosomal peptide synthetase adenylation domains**. *Chem & Biol* 2000, **7**(3):211-224.
10. Walton JD, Panaccione, DG, and Hallen HE.: **Peptide Synthesis without Ribosomes**. In: *Advances in Fungal Biotechnology for Industry, Agriculture, and Medicine*. JS Tkacz and LLange, Eds. New York, New York: Kluwer Academic/Plenum Publishers; 2004, 127-162.
11. Tudzynski P, Correia T, Keller U: **Biotechnology and genetics of ergot alkaloids**. *Appl Microbiol and Biotechnol* 2001, **57**(5-6):593-605.
