# Supplementary material for: Module evolution and substrate specificity of fungal nonribosomal peptide synthetases involved in siderophore biosynthesis
Source: BMC Evol Biol. 2008 Dec 3;8:328. doi: 10.1186/1471-2148-8-328 (PMC2644324; doi:10.1186/1471-2148-8-328)
Supplement: Additional file 6 — Additional file2, 3, 4figure legends. Figure legends for additional files 2, 3, 4. [file 1471-2148-8-328-S6.pdf]

Additional File Figure Legends

Additional file 2.
Species tree.

Tree used for reconciliation analyses was adapted from four recent phylogenetic studies [32][29-31]. Dothideomycete taxa were placed as sister to other filamentous ascomycetes in the subphylum Pezizomycotina (see Materials and Methods).

Additional file 3.
Bayesian analyses of all A domains examined in this study.

As with the ML analysis (Fig. 3), N-terminal A domains of both lineages group together and C-terminal domains of both lineages group together (thick vertical bars). NPS2, module 2 groups with the C-terminal modules, while NPS1/SidC module 2 and Dothideomycete NPS2 module D.3 group with the N-terminal modules. See Fig. 3 for numbered node descriptions, species and Accession numbers, and nomenclature used. Bayesian posterior probabilities are indicated above branches. Note that in the Bayesian tree, the A domains of SidE module 1 group as in the ML tree (Fig. 3).

Additional file 4.
Individual NPS2 and NPS1/SidC A domain lineage analyses.

A-B. Maximum likelihood (i) and Bayesian (ii) analyses of A and C domains, respectively, of ferrichrome synthetases in the NPS2 lineage.

A. AMP domains. In both trees, A domains of module 2 group with those of C-terminal module 3 or 4 while A domains of Dothideomycete module D3 groups with those of N-terminal module 1. Bootstrap and posterior probability support respectively for these relationships are shown above branches. A domains of sib1 modules 1 and 3, group with other N and C- terminal module A domains, respectively. The degenerate A domain of sib1 module 2, varies in placement. Only module 1 and module 3 of *H. capsulatum* are included as the module 2 and 6 A domains are missing due to poor sequence quality.

B. CON domains. In (i) and (ii) trees, C domains of modules 6 and 4 and those of module 5 or 3 group together. Bootstrap and posterior probability support for these relationships are shown above branches. The C domain of module 2 groups with modules 6 and 4 in the ML tree, but is unresolved in the Bayesian tree. Note, as indicated in the text, some SidE proteins have a N-terminal C domain. Here, for all SidEs, we used the C domain from the first complete (A-T-C) module. C domains of sib1 modules 3 and 6, group with the corresponding C domains of other NPS2 members, however all other sib1 C domains vary in placement. Only four C domains (C1, C3-5) of the *H. capsulatum* gene are shown as C2 and C6 are missing due to poor sequence quality.

C-D. Maximum likelihood (i) and Bayesian (ii) analysis of A and C domains, respectively, of ferrichrome synthetases in the NPS1/SidC lineage.

C. AMP domains. In both trees, A domains of module 1 and 2 group together while those of module 3 group separately. Bootstrap and posterior probability support respectively are shown above branches. *U. maydis* has two ferrichrome synthetases, fer3 and sid2. fer3 domains clearly group with the corresponding domains of the majority of the members of this lineage. *U. maydis* sid2 module 1 C domain, consistently groups with the module 2 C domains of the

majority of the members of this lineage, while C domains of both sid2 modules 2 and 3 group with other module 3 C domains. In both trees, it is clear that *U. maydis* sid2 domains group separately from the fer3 domains, supporting the hypothesis of a duplication within this lineage. The A domains of FG11026 and CHG02251 clearly group separately from other ascomycete genes within this lineage supporting additional duplication within this lineage.

D. CON domains. In (i) and (ii) trees, C domains of modules 6 and 4 and those of module 5 or 3 group together. Bootstrap and posterior probability support for these relationships respectively are shown above branches. The C domain of module 2, varies in placement while the C domain of module 1 also appears sister to the SidE outgroup.
